# Biocatalytic structural diversification of saponins in *Zygophyllum decumbens*: HR-LC-ESI-TOF-MS/MS profiling and bioactivity modulation

**DOI:** 10.1186/s12906-026-05377-4

**Published:** 2026-04-25

**Authors:** Nagwan M. Gabr, Soad M. Abd-ElKhalik, Jaky T. Zaki, Lina J. M. Abdel-Hafez, Alaadin E. El-Haddad

**Affiliations:** 1 Pharmacognosy Department, Faculty of Pharmacy, Capital University (formerly Helwan University), Cairo, 11795 Egypt; 2 Pharmacognosy Department, Faculty of Pharmacy, October 6 University, Giza, 12585 Egypt; 3 Microbiology & Immunology Department, Faculty of Pharmacy, October 6 University, Giza, 12585 Egypt

**Keywords:** *Zygophyllum decumbens*, Triterpenoid saponins, Biocatalysis, Antioxidant, Antifungal, Cytotoxicity

## Abstract

**Background:**

*Zygophyllum decumbens* has been traditionally used in the treatment of gout, fungal infections, diabetes, and rheumatism. These therapeutic effects are primarily attributed to its triterpenoid saponins (TS). The present study explores the biocatalytic transformation of TS to examine structural modifications, identify rare saponins, and evaluate associated changes in biological activity.

**Methods:**

Triterpenoid saponins were isolated from shade-dried aerial parts by cold maceration in aqueous alcohol, followed by fractionation and precipitation with acetone, yielding (TS). Three microbial strains, *Aspergillus niger*, *Lactobacillus acidophilus*, and *Bacillus subtilis*, were evaluated for their biocatalytic potential. *A. niger* demonstrated the highest efficiency and was therefore selected for large-scale biocatalytic transformation, producing a modified saponin fraction (TSM). Both TS and TSM were characterized using high-resolution LC-ESI-TOF-MS/MS. Antifungal activity was assessed by agar diffusion and minimum inhibitory concentration (MIC) assays. Cytotoxic effects were determined using the MTT assay on HepG-2, HT-29, and BNL-CL2 cell lines, while antioxidant activity was evaluated using DPPH and ABTS radical scavenging assays.

**Results:**

A total of thirty-three saponins were tentatively identified, with quinovic acid derivatives predominating. Biocatalysis led to the formation of eight new rare saponins and the loss of two pre-existing ones. Both TS and TSM demonstrated selective antifungal activity, with TSM producing broader inhibition zones and over 70% MIC reduction in most strains, except against *Penicillium marneffei*. Both fractions exhibited weak cytotoxicity (IC_50_ > 300 µg/mL) against the tested cell lines. Antioxidant activity declined post-biocatalysis as DPPH values dropped from 40.91 ± 1.87 to 19.90 ± 0.22 µM Trolox equivalents/mg, and ABTS values from 309.66 ± 11.10 to 165.78 ± 4.62 µM Trolox equivalents/mg.

**Conclusion:**

Biocatalytic treatment with *A. niger* caused pronounced structural modifications in *Z. decumbens* saponins, resulting in enhanced selective antifungal activity accompanied by diminished antioxidant capacity. These results highlight microbial biocatalysis as an effective approach for generating structural diversity in plant-derived compounds and modulating their pharmacological properties.

**Graphical Abstract:**

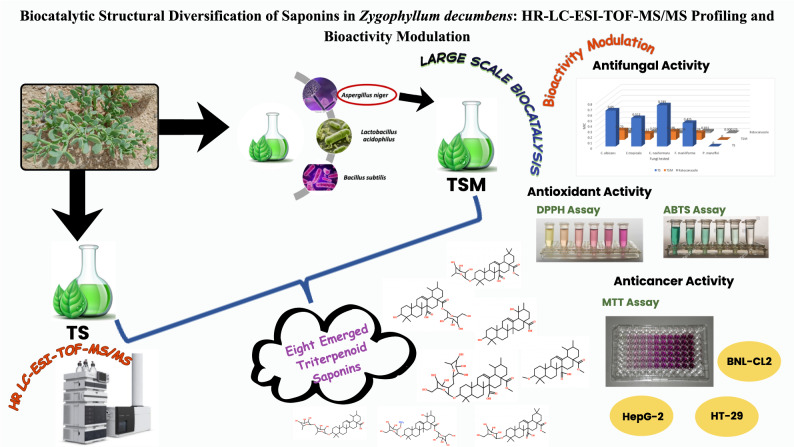

**Supplementary Information:**

The online version contains supplementary material available at 10.1186/s12906-026-05377-4.

## Background

Triterpenoid saponins are a naturally occurring class of glycosides widely distributed across various plant families, including Fabaceae, Araliaceae, Caryophyllaceae, Sapindaceae, and Zygophyllaceae [1]. Triterpenoids are chemically defined as oxygenated forms of triterpenes, usually featuring either pentacyclic or tetracyclic frameworks. Among the pentacyclic triterpenoids, the most common structural types include lupane, oleanane, and ursane, all built from six isoprene units [2]. These compounds are noted for broad pharmacological effects, as triterpenoid saponins display antioxidant, anti-inflammatory, antimicrobial, hepatoprotective, anticancer, and antidiabetic activities [3]. The diversity of their bioactivities makes them attractive scaffolds for designing novel therapeutic agents to manage a range of human diseases.

In recent years, increasing attention has been directed towards the contribution of reactive oxygen and nitrogen species (ROS/RNS) in oxidative processes within biological systems. These species include both highly reactive free radicals and non-radical molecules [4]. Under normal physiological conditions, ROS/RNS production and elimination are tightly regulated; disruption of this balance leads to oxidative stress, which contributes to diseases such as Alzheimer’s disease, neurodegenerative disorders, diabetes, and cardiovascular conditions. Accordingly, growing interest has focused on natural antioxidants as safer, less toxic alternatives to synthetic agents [5, 6].

Beyond their antioxidant properties, triterpenoid saponins show notable cytotoxicity against multiple human cancer cell lines, including breast (MCF-7), cervix (HeLa), colon (HT-29), and liver (HepG-2) cell lines [7, 8]. In addition to having been reported to possess potent antimicrobial activities, particularly against a range of pathogenic fungi [9]. These properties enhance their appeal as multifunctional agents in drug discovery.

Although therapeutically promising, triterpenoid saponins pose drug-development challenges because of poor solubility and limited bioavailability. Generally, saponins are poorly absorbed in the human gastrointestinal tract. However, rare triterpenoid saponins, often derived from the hydrolysis or modification of glycosylated moieties, have demonstrated markedly enhanced bioavailability and improved pharmacological profiles. Consequently, converting common saponins into their corresponding rare counterparts has attracted growing interest in pharmaceutical and nutraceutical research. Various methods have been employed to achieve this transformation, including physical treatments (e.g., thermal processing), chemical hydrolysis (e.g., acid-mediated cleavage), and microbial biocatalysis. Among them, microbial biocatalysis is particularly favored for the large-scale production of rare, bioactive saponins due to its high selectivity, environmentally friendly conditions, and minimal formation of undesirable byproducts [10–13].

Among the microbes studied, *Aspergillus niger* (*A. niger*) stands out for its versatility in both laboratory and industrial scale biocatalysis [10, 14, 15]. It is generally recognized as being safe for use in food and pharmaceutical applications [16]. *A. niger* can perform a range of targeted chemical transformations in triterpenoid saponins, including hydroxylation, oxidation, reduction, rearrangement, hydrolysis, epimerization, and isomerization, with high selectivity [11, 12].

The family Zygophyllaceae includes 27 genera and around 285 species, with *Zygophyllum* being the largest genus, comprising nearly 80 species [17]. A review of current literature highlights numerous studies reporting the presence of beneficial phytochemicals among members of this genus, particularly triterpenoid saponins [18, 19]. For example, *Z. album* and *Z. cocceniem* are reported to be rich with various quinovic acid glycosides [20, 21], which are thought to contribute significantly to both their antioxidant, antifungal, and cytotoxic activities against HepG-2 and PC3 cell lines [21, 22].

So far, reports on triterpenoid saponins from *Zygophyllum decumbens* (*Z. decumbens*) are limited to the isolation of three compounds: zygophylosides I, J, and K [23]. However, no studies have yet explored the total triterpenoid saponin profile or the biological modulation in *Z. decumbens* following microbial biocatalysis.

In this study, the crude triterpenoid saponin fraction of *Z. decumbens* was biocatalyzed using three microbial strains. *A. niger* showed the highest transformation efficiency and was used for large-scale processing. A comparative analysis of saponins before and after biocatalysis (TS and TSM, respectively) focused on changes in their chemical composition and biological activity. High-resolution LC-ESI-TOF-MS/MS was used to profile their chemistry. Antifungal activity was tested using agar diffusion and minimum inhibitory concentration (MIC), cytotoxicity against liver cancer (HepG-2), colon cancer (HT-29), and normal mouse liver (BNL CL.2) cell lines *via* MTT assay, and antioxidant capacity using DPPH and ABTS assays.

## Materials and methods

### Plant material

**Fig. 1:**
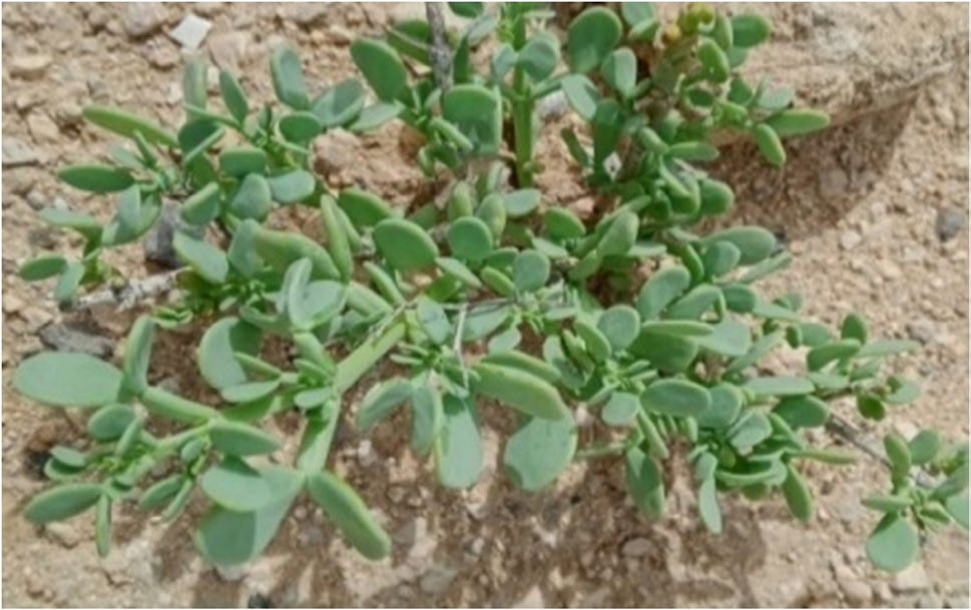
Aerial parts of *Zygophyllum decumbens* (X = 0.06)

The aerial parts of *Zygophyllum decumbens* (Fig. 1) were collected from Wadi Hagul (Cairo-Suez Road) in April 2022 with authorization from the Agricultural Research Centre, Giza. Taxonomic identification was carried out by Dr. A. Abd-Elmogali of the Flora and Phyto-taxonomy Department, Horticultural Research Institute, Agricultural Research Centre (ARC), Giza, Egypt. The collection followed institutional, national, and international guidelines, with all necessary permits secured. A voucher specimen was authenticated and deposited in the Herbarium of the Flora and Phyto-taxonomy Department, Horticultural Research Institute, Agricultural Research Centre (ARC), Giza, Egypt, under voucher number 4752 (CAIM).

### Chemicals

All chemicals used were of analytical grade, including DMSO, potassium persulfate, methanol, ethyl acetate, ethanol, ammonium hydroxide, vanillin-sulfuric acid, n-butanol, acetone, phosphate Tris buffers, DPPH, and ABTS. Ketoconazole, doxorubicin, DMEM, SDB, and MTT were purchased from Sigma-Aldrich. Unless stated otherwise, general lab reagents were sourced from Sigma-Aldrich (St. Louis, MO, USA) and Merck Millipore (Darmstadt, Germany).

### Metabolite extraction

One kilogram of shade-dried aerial parts of *Z. decumbens* was extracted by cold maceration with 70% aqueous methanol (7 × 3 L) at room temperature. The combined extracts were filtered through Whatman No. 1 filter paper, and the solvent was evaporated under reduced pressure at 40 °C using a rotary evaporator (Buchi, AG, Switzerland) to obtain 142 g of dry crude extract. A portion of the crude extract (70 g) was dissolved in 300 mL of distilled water and successively partitioned with methylene chloride (3 × 200 mL), ethyl acetate (3 × 200 mL), and *n*-butanol (3 × 200 mL). The *n*-butanol fraction was concentrated under reduced pressure at 40 °C to yield 10.5 g of dry extract, which was dissolved in 10 mL of methanol and precipitated with acetone (3 × 250 mL). This process afforded 8.3 g of a dried precipitate, designated as TS. The remaining 72 g of the crude extract was subjected to microbial biocatalysis; 2 g was used for microorganism screening, while the remaining 70 g was employed for large-scale biocatalytic transformation. Following biocatalysis, the extract was processed using the same partitioning and precipitation procedures, yielding 3.5 g of dried material, designated as TSM.

### Microbial biocatalysis

#### Microorganisms

Microorganisms were obtained from the Microbiological Resources Centre (MIRCEN) at Ain Shams University, Cairo, Egypt. They were stored in Sabouraud Dextrose Broth at 4 °C. The screening for triterpenoid saponin biocatalysis was conducted using three strains: *Aspergillus niger* (ATCC 10404), *Lactobacillus acidophilus* (ATCC 4356), and *Bacillus subtilis* (ATCC 6051).

#### Microorganisms screening procedures

All three strains were grown in 100 mL of Sabouraud Dextrose Broth (SDB) at 25 ± 2 °C for 14 days [24]. Each culture was then standardized to 10⁷ CFU/mL in phosphate-buffered saline (PBS) containing Tween-20 (5 µL/mL) [25] and incubated at 28 °C with shaking (180 rpm) for 48 h. A 5 mL aliquot of each pre-culture was transferred to 30 mL of fresh SDB (pH adjusted to 5.0 before sterilization) for propagation. For biotransformation, 0.6 g of alcohol plant extract in 2 mL of distilled water was added to 25 mL of inoculated SDB per strain. Cultures were monitored by sampling ~ 1 mL after 24 h and then daily for two weeks. Samples were extracted *via* liquid-liquid partitioning as previously described and analysed by TLC using silica gel 60 GF254 plates with a butanol: ethanol: ammonia (7:2:5) system, visualized with vanillin-sulfuric acid. Culture and substrate controls were run for comparison.

#### Large-scale biocatalysis and isolation of metabolites

Among the three screened microorganisms, *Aspergillus niger* (ATCC 10404) was chosen for large-scale biocatalysis due to its consistent and reproducible production of metabolites, as shown in Figure 1S. For the biocatalysis experiment, 70 g of the methanol plant extract was suspended in 20 mL of distilled water and added to 200 mL of SDB medium inoculated with *A. niger* spores. The mixture was incubated for 14 days under continuous shaking at 180 rpm, with the progress of biocatalysis monitored during the incubation period [26]. Upon completion, the culture was filtered, and the resulting broth was fractionated as previously described, yielding 3.5 g TSM.

### HR LC-ESI-TOF-MS/MS analysis

The phytochemical profiles of both TS and TSM were analysed using high-resolution liquid chromatography coupled with electrospray ionization time-of-flight tandem mass spectrometry (HR LC-ESI-TOF-MS/MS), as described by [27]. For analysis, 50 mg of each fraction was dissolved in 1 mL of a water: methanol: acetonitrile (50:25:25, v/v) mixture, sonicated for 10 min, and centrifuged at 10,000 rpm for 10 min. A 50 µL aliquot was diluted to 1 mL with the same solvent, and 10 µL was injected at room temperature into the LC-MS system in both positive and negative ionization modes; blanks served as controls. Chromatographic separation (28 min, 0.3 mL/min) was performed on an ExionLC system (AB Sciex, USA) using an XSelect HSS T3 column (2.5 μm, 2.1 × 150 mm; Waters) at 40 °C, with 0.5 μm × 3.0 mm Phenomenex^®^ filters and autosampler. The gradient: 100% A (1 min), linear to 10% A (20 min), held (4 min), returned to 100% A (1 min), and re-equilibrated (3 min). Mobile phase A was 5 mM ammonium formate (pH 3.0 for positive, pH 8.0 for negative ion mode) with 1% methanol; B was 100% acetonitrile. Mass spectrometry was conducted on a TripleTOF™ 5600+ (AB Sciex) with a Duo-Spray ESI source. Ionization used ± 4500 V spray voltage, ± 80 V de-clustering voltage, 500 °C source temp, curtain gas at 25 psi, and gases 1 and 2 at 45 psi. Data were acquired in IDA mode, capturing full-scan MS and MS/MS spectra. Collision energy was ± 35 V with a 20 V spread; ion tolerance was 10 ppm. A 0.6502 s survey scan triggered MS/MS for the top 15 ions (50–1100 m/z).

### Data analysis and compound annotation

Raw data were processed using MS-DIAL version 4.9 (https://zenodo.org/records/12589462), and PeakView v1.2 (https://www.peakviewer.com) was employed to extract peaks from the total ion chromatograms (TIC). Tentative metabolite identification was based on comparisons of retention times, exact m/z values, and MS/MS fragmentation patterns with existing reports in the literature and referenced in MassBank, FoodDB, and ReSpect databases.

### Determination of antifungal activity

Fungal strains, including *Candida albicans* (ATCC 10231), *Candida tropicalis* (RCMB 005004), *Cryptococcus neoformans* (RCMB 0049001), *Fusarium moniliform* (RCMB 008005), and *Penicillium marneffei* (RCMB 001022), were obtained from the Regional Centre for Mycology and Biotechnology, Al-Azhar University. All microorganisms were cultivated on SDA (HiMedia Laboratories, USA) at 25 °C for 48 h. A single colony from each strain was subsequently inoculated into 2 mL of Sabouraud Broth and incubated overnight under the same conditions.

#### Cup agar diffusion method

Antifungal activity of TS and TSM was determined through the agar cup diffusion technique [28] in comparison with ketoconazole. Fungal suspensions were standardized to an optical density (OD) of 0.5 at 600 nm. Plates of Sabouraud Dextrose Agar (SDA) with 100 µL of the respective fungal cultures. Agar wells with a diameter of 5 mm were punched into the medium and filled with 100 µL of the tested solutions, prepared in sterile water at a concentration of 5 mg/mL. Plates were incubated at 25 °C for 48 h for all fungal strains. Antifungal efficacy was assessed by measuring the inhibition zone diameter (in millimetres). All assays were performed in triplicate, and results were expressed as mean ± standard deviation (SD).

#### Broth microdilution method

This method was employed to assess the antifungal susceptibility of both TS and TSM. Two-fold serial dilutions were prepared in Mueller-Hinton Broth to achieve a concentration range of 2.5 to 0.078 mg/mL in a 96-well microtiter plate. Each well was inoculated with a fungal suspension of *C. albicans*, *C. tropicalis*, *C. neoformans*, *F. moniliform*, and *P. marneffei* prepared by diluting a standardized inoculum (adjusted to 0.5 McFarland standard) in the same medium. The plates were incubated under appropriate conditions for each fungus, as previously described by [29]. MIC was determined as the minimum concentration of the extract that entirely prevented visible fungal growth and was documented for each fungus.

### Determination of cytotoxic activity

The cytotoxic potential of both TS and TSM was assessed in comparison with doxorubicin, serving as the reference standard, using the MTT assay [30, 31]. The cytotoxicity study was performed on HepG-2 (human liver cancer), HT-29 (colorectal cancer), and BNL CL.2 (normal mouse liver) cell lines, obtained from Nawah Scientific Inc. (Cairo, Egypt). Test fractions were assessed across concentrations ranging from 0.03 to 300 µg/mL. Cells (5 × 10ⁿ/well; *n* = 3) were seeded in 96-well plates with 190 µL of medium and incubated at 37 °C, 5% CO₂ for 24 h, followed by 18 h in drug-free medium for attachment. Samples (10 µL; DMSO: water, 1:9) were added, and cells were incubated for 48 h. MTT solution (10 µL, 5 mg/mL) was then added and incubated for 4 h, followed by 100 µL SDS-HCl and a further 4 h incubation. Absorbance at 570 nm was recorded using a FLUOstar Omega reader. Cell viability and IC₅₀ values were calculated. All assays were triplicated; data are presented as mean ± SD.

### Determination of antioxidant activity

Antioxidant potential of TS and TSM was determined using DPPH and ABTS assays. A 2 mM Trolox stock solution was prepared in methanol and then subjected to serial dilution to generate a series of concentrations. The test samples were first dissolved in DMSO at 50 mg/mL, then further diluted with methanol to obtain a final working concentration of 1 mg/mL.

#### DPPH free radical scavenging assay

In a 96-well plate (*n* = 3), 100 µL of each of TS and TSM was combined with 100 µL of a freshly prepared 0.1% DPPH methanolic solution. Following incubation in the dark at 25 °C for 30 min, absorbance was measured at 540 nm using a FluoStar Omega microplate reader [32].

#### ABTS free radical scavenging assay

ABTS (192 mg) was dissolved in 50 mL of distilled water. To generate the radical cation, 1 mL of this solution was mixed with 17 µL of 140 mM potassium persulfate and incubated in the dark for 24 h. The resulting mixture was then diluted with methanol to a final volume of 50 mL to prepare the working ABTS solution. For the assay, 190 µL of the working solution was dispensed into each well of a 96-well plate (*n* = 3), followed by the addition of 10 µL of the test sample. The plate was incubated in the dark at 25 °C for 30 min, and the decrease in absorbance was recorded at 734 nm using a FluoStar Omega microplate reader [32].

### Statistical analysis

Results are presented as mean ± standard deviation (SD). Before statistical analysis, datasets were examined to ensure the fulfillment of one-way analysis of variance (ANOVA) assumptions by assessing normality using the Shapiro–Wilk test and equality of variances using Levene’s test. Upon the confirmation that the required assumptions were met, statistical comparisons were carried out using ANOVA, followed by Tukey’s multiple comparisons post hoc test to identify significant differences among the evaluated extracts. A p-value of < 0.05 was considered statistically significant. All analyses and graphical representations were conducted using GraphPad Prism software version 9 (GraphPad Software, San Diego, CA, USA). Groups marked with different letters denote statistically significant differences.

## Results

### Metabolic profiling

**Fig. 2:**
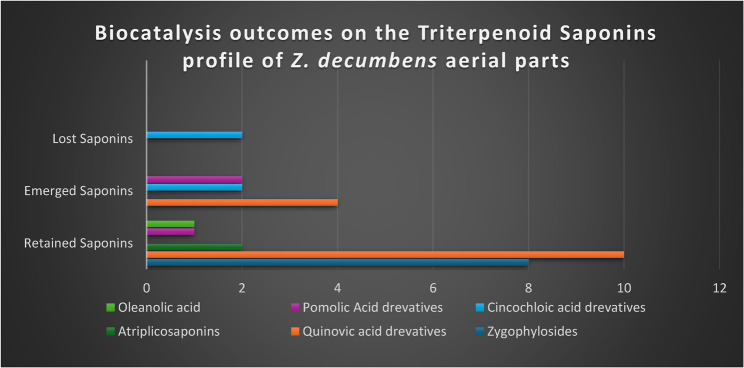
Biocatalysis outcomes on the crude triterpenoid saponins profile of *Z. decumbens* aerial parts

The metabolites present in TS and TSM of *Z. decumbens* aerial parts were analysed using High-resolution LC-ESI-TOF-MS/MS in conjunction with relevant databases in an attempt to monitor the impact of microbial biocatalysis. The detected metabolites were tentatively identified by comparing their chromatographic profiles, retention times (Rt), *m/z* values in total ion chromatograms (TIC) under both negative and positive ionization modes, as well as their fragmentation patterns, with those reported in the literature and listed in MassBank, the FoodB Database, and the ReSpect database for plant-derived compounds. Tables 1S and 2 S, and Fig. 2S, and 3S display twenty-five triterpenoid saponin metabolites identified before microbial biocatalysis (TS), among which quinovic acid–based derivatives were the dominant compounds. Following microbial biocatalysis, thirty-one triterpenoid saponin metabolites were tentatively detected in TSM, also with quinovic acid derivatives as the major compounds. Microbial biocatalysis resulted in the formation of eight rare saponins, whereas the remaining metabolites were unchanged. As detailed in Tables 1S and 2S, and Figs. 4-36 S, twenty-three of these triterpenoid saponins were found in both TS and TSM. Notably, cincholic acid-*O*-glucoside [21] and cincholic acid-*O*-quinovoside [29] were detected exclusively in TS. In contrast, eight rare saponins were unique to TSM Figs. 4-11 S, including 28-*O-β-*D-glucoside ester of pomolic acid 3-*O-β-*D-2-O-sulfonyl-glucoside [9], methyl ester of quinovic acid-*O*-glucosyl-rhamnoside [10], quinovic acid-*O*-hexosyl ester [16], methyl ester of cincholic acid-*O*-glucoside [19], methyl ester of 3-*O*-methoxy quinovic acid [28], methyl ester of cincholic acid-*O*-quinovoside [30], pomolic acid [32], and 14-decarboxyquinovic acid-*3β-O-β-*D-quinovosyl-(1→4)-quinovoside [33].

### Evaluation of antifungal activity

Table 1 reveals that TSM consistently exhibited superior antifungal efficacy compared to TS, as evidenced by its larger IZ values and significantly lower MICs across all susceptible strains. Notably, TSM showed the strongest activity against *C. albicans* and *C. tropicalis* with an inhibition zone (IZ) of 31 mm and 29 mm, and a MIC of 0.172 mg/mL and 0.112 mg/mL, respectively. While ketoconazole demonstrated the lowest MIC values overall, indicating its high potency, TSM displayed comparable or even larger IZ in some cases, suggesting effective surface-level inhibition. However, both TS and TSM showed no activity against *P. marneffei.*

**Table 1:** Evaluation of the antifungal activity of TS *versus* TSM extract of *Z. decumbens* aerial parts

Fungi tested	TS extract		TSM extract		Ketoconazole	
	IZ (mm)	MIC (mg/mL)	IZ (mm)	MIC (mg/mL)	IZ (mm)	MIC (mg/mL)
*C. albicans*	28 ± 0.52^a^	0.650	31 ± 0.58^b^	0.172	25 ± 0.42^a^	0.008
*C. tropicalis*	**24 ± 0.60** ^**a**^	**0.512**	29 ± 0.51^b^	0.112	26 ± 0.55^a^	0.016
*C. neoformans*	25 ± 0.48^a^	**0.745**	30 ± 0.41^b^	0.148	21 ± 0.40^c^	0.00054
*F. moniliforme*	23 ± 0.55^a^	0.425	28 ± 0.46^b^	0.124	20 ± 0.50^a^	0.031
*P. marneffei*	NA	NA	NA	NA	23 ± 0.65	0.000125

*IZ *Inhibition zone is expressed as mean ± Standard Deviation (SD), where *n* = 3 in three independent assays. *NA *Not active

Values marked with different letters denote statistically significant differences with *P* < 0.05

### Evaluation of cytotoxic activity

**Fig. 3:**
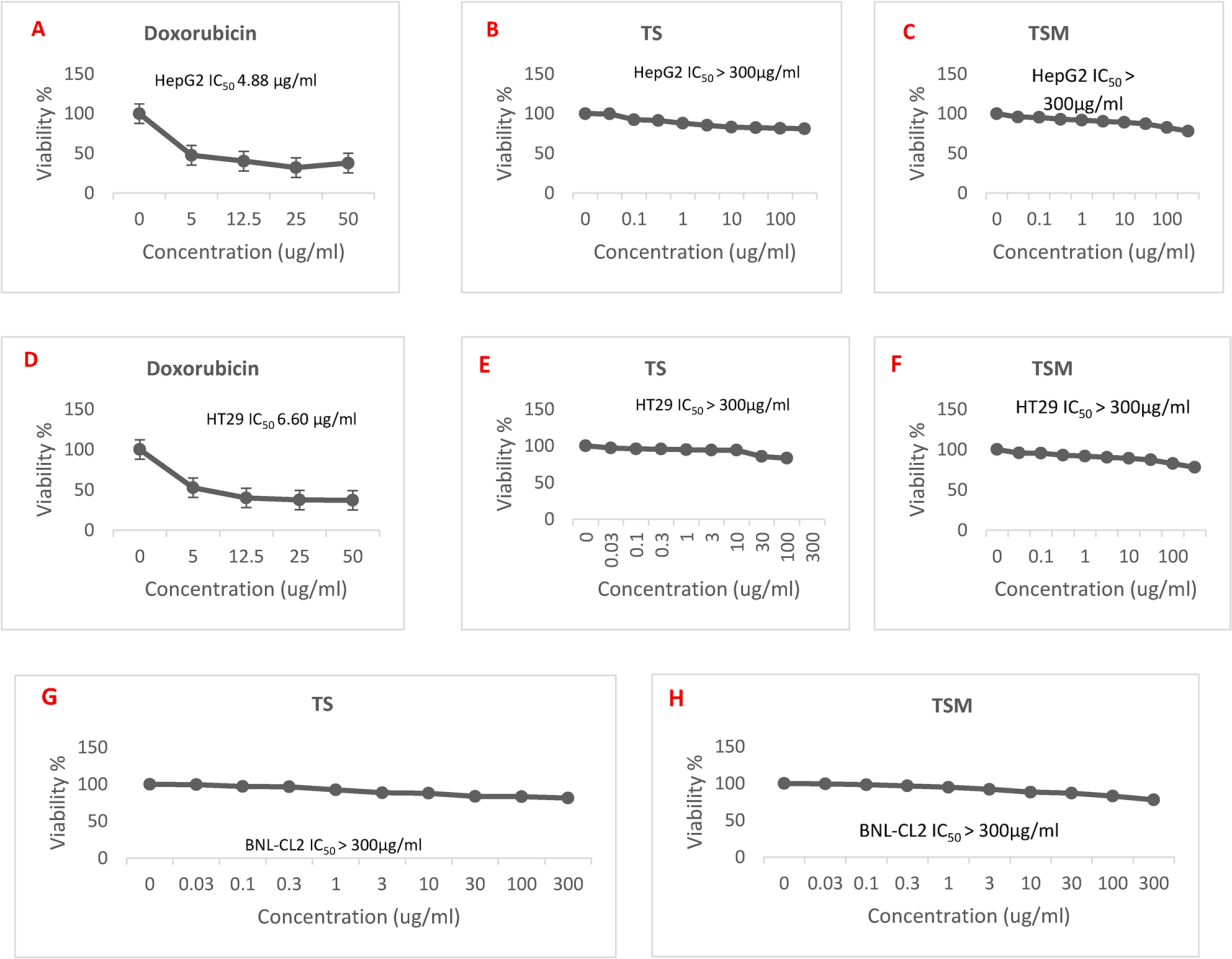
**A**-**H**: Cytotoxic evaluation of TS & TSM of *Z. decumbens* aerial parts on HepG2, HT29 & BNL-CL2 cell lines data are presented as mean ± standard deviation (SD) (*n* = 3)

Figures 3(A-H) & 37S reveal that TS and TSM can be described as non-cytotoxic, as their IC₅₀ values exceeded 300 µg/mL for all the tested cell lines, as per the recommendations of the National Cancer Institute (USA, NCI), a plant fraction showing an IC₅₀ value < 20–30 µg/mL is considered a cytotoxic drug [33]. In contrast, doxorubicin exhibited potent cytotoxicity with IC₅₀ values of 4.88 µg/mL and 6.60 µg/mL against HepG2 and HT29, respectively. These findings suggest that TS and TSM have no significant cytotoxicity under the tested conditions.

### Evaluation of antioxidant activity

**Fig. 4:**
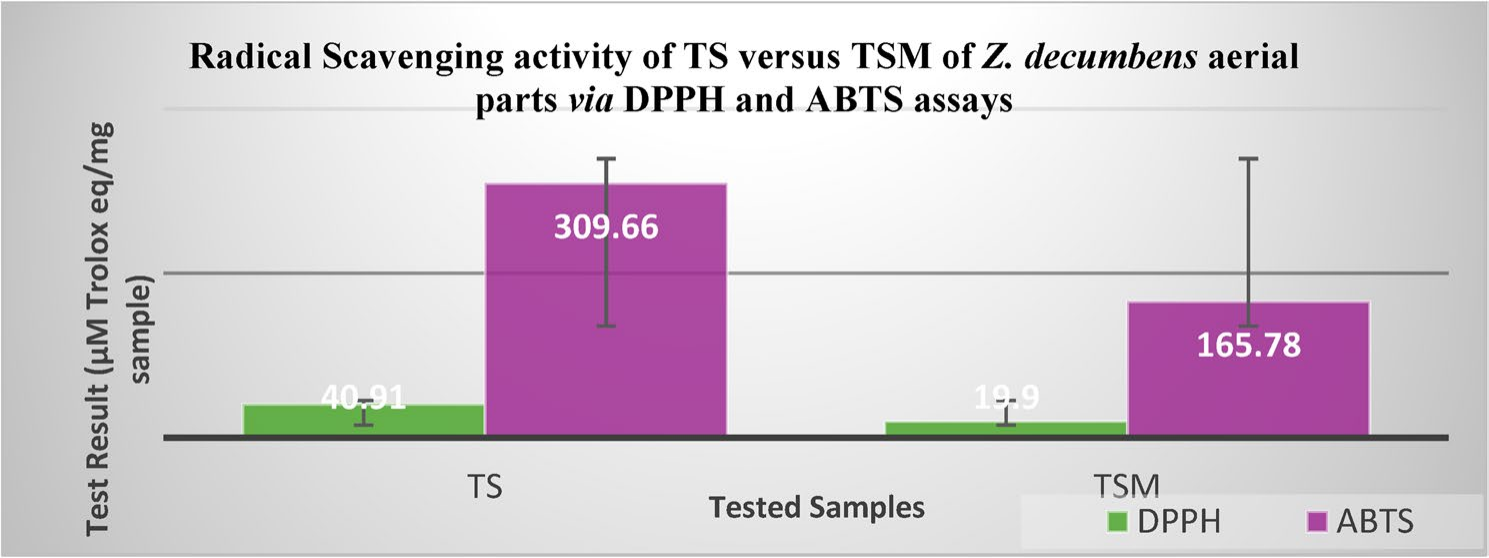
The antioxidant activity of TS *versus* TSM of *Z. decumbens* aerial parts *via* DPPH and ABTS assays. Results are represented as µM Trolox equivalents per mg of sample ± Standard Deviation (SD), where n = 3 in three independent assays

Findings in Fig. 4 indicate a substantial reduction in antioxidant potential following microbial biocatalysis, as TS achieved significantly higher antioxidant efficacy in both DPPH and ABTS assays, exhibiting nearly a twofold increase compared to TSM.

## Discussion

The triterpenoid saponin profile of *Z. decumbens* was characterized using LC-MS/MS in both negative and/or positive ionization modes, providing informative fragmentation patterns crucial for structural elucidation (Table 1S). The detected compounds displayed characteristic neutral losses of one or more sugar moieties, such as hexosyl (162 Da), deoxyhexosyl (146 Da), and hexouronyl (176 Da), alongside prominent fragment ions corresponding to different aglycone cores, like ursane and oleanane. Retro-Diels–Alder (RDA) fragmentation of the aglycone skeleton involving cleavage of ring A or C further yielded diagnostic ions typically in the *m/z* 200–350 range, aiding in the identification of the core triterpenoid structure [34–36]. The triterpenoid saponin glycosides were identified based on sequential sugar loss and the presence of stable aglycone fragment ions. For example; the deprotonated signal [M-H]^−^ of 3-*O*-[*α-*L-arabinosyl-(1→2)-*β-*D-glucosyl] quinovic acid 28-(*β-*D-glucosyl) ester [6], 3-*O*-[*β*-D-glucosyl-(1→2) *β*-D-glucosyl] quinovic acid-28-*O-β*-D glucosyl ester [7], atriplicosaponin A [13], and *O*-quinovosyl cincholic acid-*O*-glucosyl ester [18], observed at *m/z* 955.3372, 971.2844, 765.1663, and 793.3646 (calcd 955.4980, 971.4929, 765.4503 and 793. 4452 for C_48_H_76_O_19,_ C_48_H_76_O_20,_ C_41_H_66_O_13_ & C_42_H_66_O_14_), respectively, and showing corresponding fragments ions at *m/z* 793, 809, 603, & 631, matching the neutral loss of 162 Da, indicating the presence of hexosyl moiety. In addition, pomolic acid 3-*O-α-*L arabinoside [24] was detected at *m/z* 605.2244 [M + H]^+^ (calcd 605.3924 for C_35_H_56_O_8_) with a fragment ion at *m/z* 471 confirming the loss of 132 Da corresponding to the loss of pentosyl unit, whereas zygophyloside G [25] was noted at *m/z* 889.2473 [M-H]^−^ (calcd 889.3969 for C_42_H_66_O_18_S) showed a fragment ion at *m/z* 548 corresponding to the loss of sulphated disaccharide (341 Da). The identification of sulphate-conjugated saponins was supported by the observation of characteristic SO₃ (80 Da) loss, as in signals [M-H]^−^ of zygophyloside F [8], 28-*O*- *β* -D-glucoside ester of pomolic acid 3-*O- β -*D-2-*O* sulfonyl-glucoside [9], zygophyloside D [15], and zygophylosides O [26] that are observed at *m/z* 873.3089, 875.4450, 711.1773, and 697.2029 (calcd 873.4020, 875.4177, 711.3492 and 697.3336 for C_42_H_66_O_17_S, C_42_H_68_O_17_S, C_36_H_56_O_12_S & C_35_H_54_O_12_S), respectively with their corresponding daughter peak appeared at *m/z* 794, 795, 631, and 617 after loss of 80 Da. Additionally, fragmentation patterns including the elimination of CO₂ (44 Da) as observed in cincholic acid -*O*- quinovoside [29], methyl ester of cincholic acid -*O*- quinovoside [30], and oleanolic acid **(31)**, in addition to elimination of H₂O (18 Da) as observed in zygophyloside I [17], and quinovic acid [23] providing further evidence of free carboxyl groups and hydroxyl substitutions respectively.

*A. niger* exhibits diverse enzymatic activities that enable extensive structural modification of triterpenoid saponins, consistent with its established role in microbial biocatalysis [15]. In this study, the generation of eight rare triterpenoid saponin derivatives may be attributed to the combined action of enzyme classes produced by *A. niger* [10, 37–39]. The observed methyl esterification of the C-28 carboxyl group in quinovic acid-*O*-glucosyl-rhamnoside [3], cincholic acid-*O-*glucoside [21], 3-*O*-methoxy quinovic acid [27], and cincholic acid-*O*-quinovoside [29] may be explained by the activity of *O*-methyltransferases, which transfer a methyl group to carboxyl or hydroxyl groups (Fig. 38S[A]) [40]. The bioconversion of decarboxyquinovic acid*-O*-rhamnoside [14] into the diglycoside 14-decarboxyquinovic acid-3-*β-O-β-*D-quinovosyl(1→4)-quinovoside [33] likely reflects the action of UDP-glycosyltransferases, which catalyse *O*-glycosylation and thereby extend existing glycan chains (Fig. 38S[B]) [41].

Partial deglycosylation observed at the C-3 position is suggested to be due to the activity of *β*-glucosidase and *α*-L-arabinosidase, both of which are abundant in *A. niger* and are capable of selectively cleaving terminal *β*-glucosyl or *α*-L-arabinose residues. This enzymatic behaviour aligns with the conversion of 3-*O*-[*β*-D-glucosyl-(1→2)-*β*-D-glucosyl]quinovic acid-28-*O-β*-D-glucosyl ester [7] and pomolic acid 3-*O*-α-L-arabinoside [24] into quinovic acid-*O*-glucosyl ester [16] and pomolic acid [32], respectively (Fig. 38S[C]) [42]. In addition, the transformation of zygophyloside G [25] into the 28-*O-β*-D-glucoside ester of pomolic acid 3-*O-β*-D-2-*O*-sulfonyl-glucoside [9] can be attributed to sequential oxidative and decarboxylative steps. Specifically, hydroxylase likely introduces hydroxyl groups at defined positions, followed by decarboxylase-mediated removal of a carboxyl moiety (Fig. 38S[D]) [15].

Overall, these findings suggest that *A. niger* promotes triterpenoid structural diversity through coordinated enzymatic transformations, supporting its utility as a versatile biocatalyst for generating rare saponin derivatives.

TSM demonstrated superior antifungal activity against *C. albicans*,* C. tropicalis*,* C. neoformans*,* and F. moniliforme* over TS, as evidenced by its larger IZ and lower MIC value can be attributable to the presence of two newly formed powerful antifungal compounds: 28-*O-β-D*-glucoside ester of pomolic acid 3-*O-β-*D-2-*O* sulfonyl-glucoside [9] and pomolic acid [32], as reported by [2]. Structure-activity relationship (SAR) studies of ursane-type triterpenoids indicate that the free C-28 carboxyl group, 3*β*-hydroxyl, and additional oxygenated substituents significantly enhance membrane-disruptive activity toward fungal cells [43], as these groups increase amphiphilicity and promote sterol-binding on fungal membranes [44]. Pomolic acid, in particular, possesses all key functional sites associated with strong biological activity [2]. Furthermore, the introduction of polar glycosidic and sulfonyl groups at C-3 and C-28, as seen in compound [9], increases solubility while retaining the hydrophobicity of the aglycone. This structure–activity relationship (SAR), repeatedly linked to enhanced antifungal potency in triterpenoid saponins, reflects the critical balance between the hydrophilicity of the glycosidic moieties and the hydrophobicity of the pentacyclic aglycone core [44]. Such structural features provide a rationale for the stronger antifungal activity observed for TSM compared with TS.

Despite the presence of various zygophylosides, oleanolic and pomolic acid derivatives, which have been previously reported to seldom exert cytotoxic effects against HepG2 and HT-29 cell lines [45, 46], both TS and TSM exhibited non-significant cytotoxic activity within the effective IC₅₀ range. As reported by [45], enhancing the cytotoxicity of oleanolic acid derivatives requires increasing ionic character or polarity at C-28, since cytotoxicity depends heavily on C-28 accessibility and the ability of the triterpenoid to interact with the cells’ lipid bilayers. The triterpenoids in TS and TSM, however, are predominantly neutral glycosides, not ionic derivatives, which are known to reduce the membrane lytic activity at biologically relevant concentrations. This trend is consistent with the SAR of triterpenoid saponins, where increasing glycosylation and esterification, especially multi-sugar substitution, typically reduces cytotoxicity by decreasing aglycone exposure and limiting membrane insertion [45].

In addition, the strong cytotoxic activity occurs only when triterpenoids are present in high abundance and act synergistically, while diluted or structurally shielded forms display substantially weaker effects [46]. Accordingly, the low abundance and extensive glycosylation/esterification of triterpenoid saponins in TS and TSM likely account for the absence of detectable cytotoxic activity.

The detection of quinovic acid derivatives such as quinovic acid-*O*-glucosyl-rhamnoside [3] and quinovic acid [25], which serve as chemomarkers for the genus *Zygophyllum*, in both TS and TSM helps explain their marked antioxidant potential [47]. In contrast, converting the free C-28 carboxyl group of triterpenoid saponins into a methyl ester has been shown to impair antioxidant performance by diminishing electron-donating capacity, a key property for neutralizing free radicals [48]. Accordingly, the appearance of four methyl-esterified triterpenoid saponins after microbial biocatalysis, methyl ester of quinovic acid-*O*-glucosyl-rhamnoside [10], methyl ester of cincholic acid-*O*-glucoside [19], methyl ester of 3-*O*-methoxy quinovic acid [28], and methyl ester of cincholic acid-*O*-quinovoside [30], may provide a rationale for the nearly 50% reduction in antioxidant activity observed in TSM relative to TS. The observed reduction in activity can be attributed to the diminished electron-donating capacity and hydrogen-bonding potential of methyl-esterified triterpenoid saponins, which in turn compromises their radical-scavenging effectiveness. However, this decline may also result from more extensive compositional alterations, such as a lower relative abundance of antioxidant saponins following biocatalytic treatment. Accordingly, the decreased activity of TSM is likely due to multiple factors.

## Conclusion

Biocatalysis by *A. niger* successfully induced targeted structural modification resulting in the formation of eight rare triterpenoid saponins. Such chemical modifications significantly enhanced antifungal activity, which can be ascribed to the production of bioactive compounds, yet their activity does not reach that of ketoconazole. In contrast, the antioxidant potential of the transformed fraction was reduced, likely due to methyl esterification that diminished radical-scavenging efficiency. Despite the presence of cytotoxic saponins, both TS and TSM exhibited weak cytotoxicity, possibly due to low active compound concentrations or antagonistic interactions within the fractions. Overall, this study underscores the potential of *A. niger*-mediated biocatalysis as an effective approach to enhance the chemical diversity and selective bioactivity of plant-derived saponins, offering promising applications in the development of targeted antifungal agents.

## Supplementary Information

Supplementary Material 1.

## Data Availability

The datasets supporting the findings of this study are included in the article and its supplementary file (Table 1S & 2S and Figures 1S to 38S).

## Abbreviations

*Z. decumbens*: *Zygophyllum decumbens*
DMSO: dimethyl sulfoxide
DPPH: 2,2-diphenyl-1-picrylhydrazyl
ABTS: 2,2′-azino-bis (3-ethylbenzothiazoline-6-sulfonic acid
DMEM: Dulbecco’s Modified Eagle Medium
MTT: 3-(4, 5-methylthiazol-2-yl)-2, 5-diphenyl tetrazolium bromide
TS: Triterpenoid saponins of Z. *decumbens* before biocatalysis
TSM: Triterpenoid saponins Z. *decumbens* post-biocatalysis
SDB: Sabouraud Dextrose Broth
SDA: Sabouraud Dextrose Agar
SD: Standard deviation
rpm: Rotation per minute
OD: Optical density
*A. Niger*: *Aspergillus niger*
HR LC-ESI-TOF-MS/MS: High-resolution liquid chromatography coupled with electrospray ionization time-of-flight tandem mass spectrometry
HepG2: Human liver cancer cell line
HT-29: Human colorectal cancer cell line
BNL CL2: Normal mouse liver cell line
UDP: Uridine diphosphate
