# Supplementary material for: Biocatalytic structural diversification of saponins in Zygophyllum decumbens: HR-LC-ESI-TOF-MS/MS profiling and bioactivity modulation
Source: BMC Complement Med Ther. 2026 Apr 25;26:155. doi: 10.1186/s12906-026-05377-4 (PMC13110427; doi:10.1186/s12906-026-05377-4)
Supplement: Supplementary file 1 — Supplementary Material 1. [file 12906_2026_5377_MOESM1_ESM.docx]

**Supplementary file**

**Table 1S: Tentatively identified metabolites in TS & TSM of *Z. decumbens* aerial parts using LC-ESI-TOF-MS/MS (Negative/ Positive modes):**

| **Triterpenoid Saponins** | | | | | | | | | | | |
| --- | --- | --- | --- | --- | --- | --- | --- | --- | --- | --- | --- |
| **No.** | **RT.**  **min** | **Tentatively Identified Metabolites** | **Formula** | **Theoretical *m/z* [M-H] ^-^** | **Theoretical *m/z* [M+H] ^+^** | **[M-H] ^-^** | **[M+H] ^+^** | **Mass Error (X10^6^)** | **Characteristic Ms/Ms** | **TS** | **TSM** |
|  | 1.01 | Zygophyloside J | C_41_H_62_O_16_ | 809.4037 | ----------- | 809.5248 | ----------- | 0.00014961 | 765,647,516,471,293 | √ | √ |
|  | 1.31 | 3-*O*- [*β*-D-(2-*O*- sulfonyl) glucosyl] quinovic acid | C_37_H_58_O_13_S | 741.3589 | ----------- | 741.0275 | 743.2824 | -0.00044696 | 499,477,400,332 | √ | √ |
|  | 1.70 | Quinovic acid-*O*-glucosyl-rhamnoside | C_42_H_66_O_14_ | 793.4452 | ----------- | 793.3758 | 795.2254 | -0.00008746 | 749,386 | √ | √ |
|  | 1.72 | Zygophylosides A | C_36_H_57_NaO_15_S_2_ | 815.3036 | ----------- | 815.2191 | 817.2858 | -0.00010364 | 635,432,355 | √ | √ |
|  | 2.64 | 3-*β*-3-[(2-*O*-sulfo-*β*-D-en glucopyranurosonyl) oxy]-urs-20 -28-oic acid 28-(2-*O*-sulfo-*β*-D-glucosyl] ester | C_42_H_68_O_19_S_2_ | 939.3796 | ----------- | 939.3693 | ----------- | -0.00001096 | 735,355 | √ | √ |
|  | 2.87 | 3-*O*-[*α*-L-arabinosyl (1→2)-*β*-D-glucosyl] quinovic acid 28- (*β*-D-glucosyl) ester | C_48_H_76_O_19_ | 955.4980 | ----------- | 955.3372 | ----------- | -0.00016828 | 937,793 | √ | √ |
|  | 2.95 | 3-*O*-[*β*-D-glucosyl-(1→2) *β*-D-glucosyl] quinovic acid-28-*O-β*-D glucosyl ester | C_48_H_76_O_20_ | 971.4929 | ----------- | 971.2844 | ----------- | -0.00021451 | 809,317,250 | √ | √ |
|  | 2.99 | Zygophyloside F | C_42_H_66_O_17_S | 873.4020 | ----------- | 873.3089 | ----------- | -0.00010659 | 793,460,385 | √ | √ |
|  | 3.04 | 28-*O*- *β* -D-glucoside ester of pomolic acid 3-*O- β -*D-2-*O* sulfonyl-glucoside | C_42_H_68_O_17_S | 875.4177 | ----------- | 875.4450 | 877.4472 | 0.00003118 | 795,534,399 |  | √ |
|  | 3.19 | Methyl ester of  Quinovic acid-*O*-glucosyl-rhamnoside | C_43_H_68_O_14_ | 807.4609 | ----------- | 807.8656 | ----------- | 0.00050120 | 400,308 |  | √ |
|  | 4.31 | Zygophylloside S | C_41_H_64_O_14_ | 779.4296 | ----------- | 779.3646 | 781.2541 | -0.00008339 | 735,386,251 | √ | √ |
|  | 4.51 | 3-*O*-arabinosyl-quinovosyl quinovic acid | C_41_H_64_O_13_ | 763.4346 | ----------- | 763.3498 | 765.2768 | -0.00011107 | 445,386,318 | √ | √ |
|  | 5.04 | Atriplicosaponin A | C_41_H_66_O_13_ | 765.4503 | ----------- | 765.1663 | 767.2932 | -0.00037102 | 747,721,603,545,288,221 | √ | √ |
|  | 5.22 | Decarboxyquinovic acid-*O*-rhamnoside | C_35_H_56_O_7_ | 587.4026 | ----------- | 587.1352 | 589.2758 | -0.00045522 | 381,261 | √ | √ |
|  | 5.41 | Zygophyloside D | C_36_H_56_O_12_S | 711.3492 | ----------- | 711.1773 | 713.2637 | -0.00024165 | 667,631,485,460,251 | √ | √ |
|  | 5.65 | Quinovic acid-*O*-glucosyl ester | C_36_H_56_O_10_ | 647.3873 | ----------- | 647.2041 | ----------- | -0.00028298 | 603,385,317 |  | √ |
|  | 5.72 | Zygophyloside I | C_42_H_66_O_16_ | 825.4350 | ----------- | 825.0517 | 827.2518 | -0.00046436 | 807,781,487 | √ | √ |
|  | 5.91 | *O*-quinovosyl cincholic acid-*O*-glucosyl ester | C_42_H_66_O_14_ | 793.4452 | ----------- | 793.3646 | ----------- | -0.00001015 | 749,631,317,250 | √ | √ |
|  | 6.04 | Methyl ester of  Cincholic acid -*O-* glucoside | C_37_H_58_O_10_ | 661.4030 | ----------- | 661.2425 | 663.3359 | -0.00024266 | 558,499,329 |  | √ |
|  | 6.16 | Atriplicosaponin B | C_42_H_70_O_14_S | 829.4468 | ----------- | 829.0902 | 831.4350 | -0.00042992 | 623,342 | √ | √ |
|  | 6.18 | Cincholic acid -*O-* glucoside | C_36_H_56_O_10_ | 647.3873 | ----------- | 647.1644 | ----------- | -0.00034430 | 603,317,251 | √ |  |
|  | 6.20 | *O-*quinovosyl quinovic acid- quinovosyl ester | C_42_H_66_O_13_ | ----------- | 779.4503 | ----------- | 779.3639 | -0.00011084 | 532,381,314 | √ | √ |
|  | 6.29 | Quinovic acid | C_30_H_46_O_5_ | 485.3345 | ----------- | 485.1666 | 487.2216 | -0.00034594 | 467,251,168,99 | √ | √ |
|  | 6.30 | Pomolic acid 3-*O-α*-L arabinoside | C_35_H_56_O_8_ | ----------- | 605.3975 | ----------- | 605.2244 | -0.00028592 | 471,305 | √ | √ |
|  | 6.32 | Zygophyloside G | C_42_H_66_O_18_S | 889.3969 | ----------- | 889.2473 | 891.2823 | -0.00016820 | 548,480,250 | √ | √ |
|  | 6.46 | Zygophylosides O | C_35_H_54_O_12_S | 697.3336 | ----------- | 697.2029 | 699.3390 | -0.00018742 | 617,447,379,318 | √ | √ |
|  | 6.50 | 3-*O*-methoxy quinovic acid | C_31_H_48_O_5_ | 499.3501 | ----------- | 499.1805 | 501.2688 | -0.00033964 | 455,385,317,249 | √ | √ |
|  | 6.57 | Methyl ester of  3-*O*-methoxy quinovic acid | C_32_H_50_O_5_ | 513.3658 | ----------- | 513.1949 | 515.2701 | -0.00033290 | 469,249,114 |  | √ |
|  | 6.77 | Cincholic acid -*O*- quinovoside | C_36_H_56_O_9_ | 631.3924 | ----------- | 631.1686 | 633.3076 | -0.00035445 | 587,485 | √ |  |
|  | 7.12 | Methyl ester of Cincholic acid -*O*- quinovoside | C_37_H_58_O_9_ | 645.4080 | ----------- | 645.2858 | 647.3062 | -0.00018933 | 601,499,313 |  | √ |
|  | 8.33 | Oleanolic acid | C_30_H_48_O_3_ | ----------- | 457.3603 | ----------- | 457.2290 | -0.00028708 | 412,235,168 | √ | √ |
|  | 8.66 | Pomolic acid | C_30_H_48_O_4_ | 471.3552 | ----------- | 471.1894 | 473.2032 | -0.00035243 | 453,427,235,100 |  | √ |
|  | 9.63 | 14-decarboxyquinovic acid-*3β-O- β*-D-quinovosyl  (l → 4)-quinovside | C_41_H_66_O_11_ | ----------- | 735.4605 | ----------- | 735.3535 | -0.00014548 | 691,528,441,393 |  | √ |

All listed metabolites are tentatively assigned. For entries showing relatively large mass deviations, identification is mainly supported by characteristic fragment ions and literature reports of related saponins. All fragmentation was applied according to the -ve mode if available, & Highlighted compounds represent new compounds that appear after microbial biocatalysis

**Table 2S: Interpretation of triterpenoid saponins of both TS and TSM tentatively identified from *Zygophyllum decumbens* aerial parts:**

| **Tentatively identified compounds** | **Chemical structure** | **Interpretation of each compound tentatively identified** |
| --- | --- | --- |
| 1. Zygophyloside J | 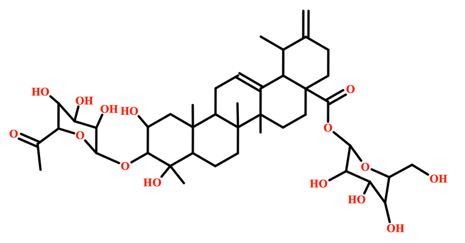 | Zygophyloside J showed a deprotonated molecular ion $\left[ M\text{–}H]^{-} \right.$at m/z 809 in the negative ion mode. The Ms/Ms spectrum revealed stepwise fragmentation, characterized by decarboxylation to m/z 765, subsequent loss of a hexose moiety yielding m/z 647, and complete deglycosylation to produce the aglycone ion at m/z 471. In addition, the fragment ion observed at m/z 293 was attributable to a retro-Diels–Alder cleavage. Collectively, these fragmentation behaviours are consistent with a triterpenoid saponin bearing a quinovic acid–type aglycone. |
|  | **Compound (1)** |  |
| 1. 3-*O*- [*β*-D-(2-*O*- sulfonyl) glucosyl] quinovic acid | 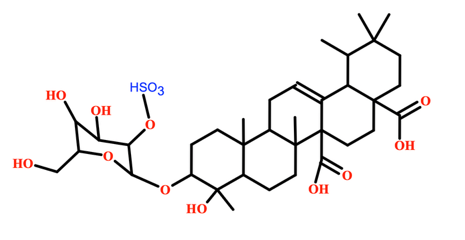 | The deprotonated molecular ion of 3‑*O*‑[*β*‑D‑(2‑O‑sulphonyl)glucosyl] quinovic acid was observed at *m/z* 741 in negative ion mode. Ms/Ms analysis showed a prominent fragment at *m/z* 499, attributable to the loss of the sulfonated hexose moiety, followed by a product ion at *m/z* 481, consistent with a further neutral loss from the aglycone. Additional fragments at *m/z* 400 and 332 were assigned to retro‑Diels–Alder cleavages within the quinovic‑acid skeleton, collectively confirming a triterpenoid core bearing a labile sulfo‑glycosidic linkage. |
|  | **Compound (2)** |  |
| 1. Quinovic acid-*O*-glucosyl-rhamnoside | 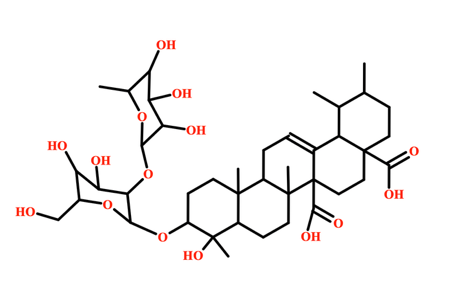 | In negative ion mode, quinovic acid‑*O*‑glucosyl‑rhamnoside exhibited a deprotonated molecular ion at *m/z* 793. The fragment at *m/z* 749 arose from the loss of the carboxylate group, consistent with decarboxylation at the C‑28 position of quinovic acid. A subsequent product ion at *m/z* 386 was attributed to sequential loss of the disaccharide moiety and CO₂, followed by retro‑Diels–Alder cleavage of the aglycone, thereby confirming the triterpenoid backbone structure. |
|  | **Compound (3)** |  |
| 1. Zygophylosides A | 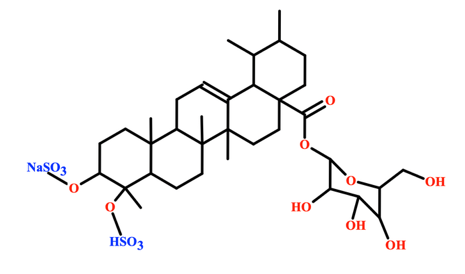 | Zygophyloside A showed a deprotonated molecular ion at *m/z* 815 in negative ion mode, consistent with a disaccharidic triterpenoid saponin. The MS/MS spectrum revealed a fragment at *m/z* 635, indicating the loss of a sugar moiety and water. Two fragments at *m/z* 432 and 355 correspond to the deglycosylated aglycone. They are characteristic of retro-Diels–Alder fragmentation within the triterpenoid core, supporting a quinovic acid-type aglycone structure. |
|  | **Compound (4)** |  |
| 1. 3-*β*-3-[(2-*O*-sulfo-*β*-D-en glucopyranurosonyl) oxy]-urs-20 -28-oic acid 28-(2-*O*-sulfo-*β*-D-glucosyl] ester | 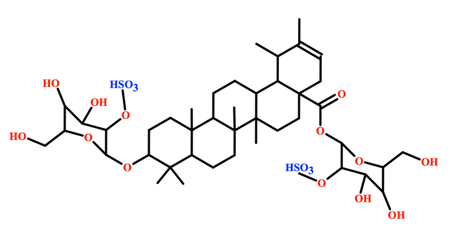 | In negative ion mode, the disulfated ursane saponin displayed a deprotonated molecular ion at *m/z* 939. The Ms/Ms spectrum showed a key fragment at m/z 735, corresponding to a combined neutral loss of 204 Da, attributable to cleavage of both sulfonate groups and the C‑28 carboxyl function. A further fragment at m/z 355 is consistent with retro‑Diels–Alder cleavage of the aglycone, confirming an urs‑20‑ene triterpenoid backbone for this saponin. |
|  | **Compound (5)** |  |
| 1. 3-*O*-[*α*-L-arabinosyl (1→2)-*β*-D-glucosyl] quinovic acid 28- (*β*-D-glucosyl) ester | 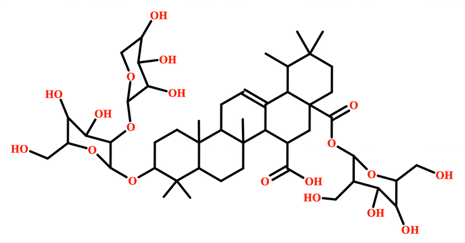 | In negative ion mode, the deprotonated molecular ion of 3‑*O*‑[*α‑*L‑arabinosyl(1→2)‑*β*‑D‑glucosyl] quinovic acid 28‑(*β*‑D‑glucosyl) ester was detected at *m/z* 955. The Ms/Ms spectrum showed a fragment at *m/z* 937, attributable to the loss of water, and a prominent product ion at *m/z* 793, corresponding to cleavage of the ester‑linked glucosyl unit at C‑28. These fragmentation features support assignment as a triglycosylated quinovic acid saponin bearing a labile glucosyl ester at position 28 |
|  | **Compound (6)** |  |
| 1. 3-*O*-[*β*-D-glucosyl-(1→2) *β*-D-glucosyl] quinovic acid-28-*O-β*-D glucosyl ester | 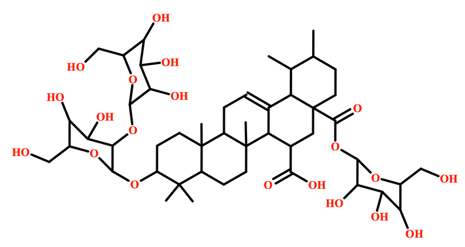 | In negative ion mode, the deprotonated molecular ion of 3‑*O*‑[*β*‑D‑glucosyl(1→2)‑*β*‑D‑glucosyl] quinovic acid 28‑*O‑β*‑D‑glucosyl ester was observed at *m/z* 971. Fragmentation produced a daughter ion at *m/z* 809, corresponding to the loss of the ester‑linked hexosyl unit at C‑28. The fragment at *m/z* 317 arises from retro‑Diels–Alder cleavage of the aglycone (rings D and E) after elimination of both sugar units and CO₂, whereas the ion at *m/z* 250 represents a deeper aglycone‑derived fragment, consistent with the triterpenoid skeleton of quinovic acid.Top of FormBottom of Form |
|  | **Compound (7)** |  |
| 1. Zygophyloside F | 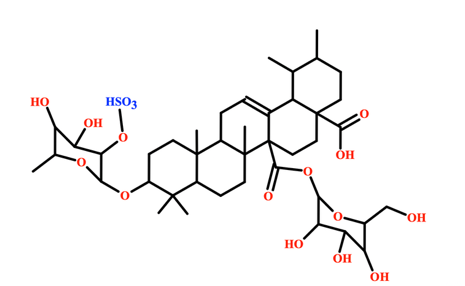 | Zygophyloside F showed a deprotonated molecular ion at *m/z* 873 in negative ion mode. A neutral loss of 80 Da yielded a fragment at *m/z* 793, indicative of a sulfate group attached to the sugar moiety. The fragment at *m/z* 460 arose from the combined loss of the sulfate, the disaccharide chain, and CO₂, exposing the aglycone core. The ion at *m/z* 385 corresponds to a retro‑Diels–Alder cleavage fragment characteristic of a quinovic acid aglycone, supporting the assignment of Zygophyloside F as a sulfated triterpenoid saponin. |
|  | **Compound (8)** |  |
| 1. 28-*O*- *β* -D-glucoside ester of pomolic acid 3-*O- β -*D-2-*O* sulfonyl-glucoside | 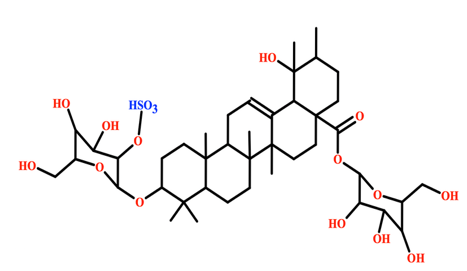 | In negative ion mode, the deprotonated molecular ion of the 28‑*O‑β*‑D‑glucosyl ester of pomolic acid 3‑*O‑β*‑D‑2‑*O*‑sulfonylglucoside was detected at *m/z* 875. The fragment at *m/z* 795 resulted from neutral loss of SO₃, confirming a sulfonylated sugar moiety. A subsequent fragment at *m/z* 534 reflected loss of both sugar units (or one sugar together with sulfate and an additional neutral loss). The low‑mass ion at *m/z* 399 was attributed to retro‑Diels–Alder fragmentation of the aglycone, supporting assignment of a pomolic‑acid triterpenoid core. |
|  | **Compound (9)** |  |
| 1. Methyl ester of Quinovic acid-*O*-glucosyl-rhamnoside | 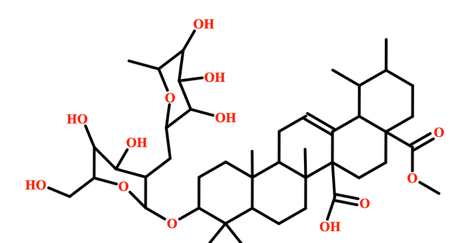 | In negative ion mode, methyl‑esterified quinovic acid‑*O*‑glucosyl‑rhamnoside exhibited a deprotonated molecular ion at *m/z* 807. The Ms/Ms spectrum showed a fragment at *m/z* 400, arising from retro‑Diels–Alder cleavage of the aglycone after deglycosylation. The ion at *m/z* 308 corresponds to the disaccharide moiety (glucosyl–rhamnoside) released upon cleavage of the glycosidic bond. |
|  | **Compound (10)** |  |
| 1. Zygophylloside S | 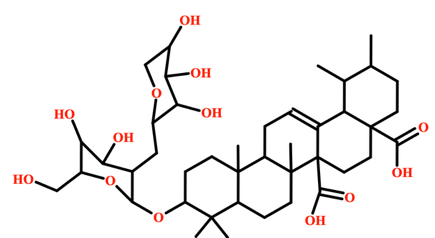 | Zygophylloside S exhibited a deprotonated molecular ion at *m/z* 779 in negative ion mode. The daughter ion at *m/z* 735 was observed due to decarboxylation of the triterpenoid’s C-28 carboxylic acid group. Subsequent fragmentation yielded a characteristic aglycone ion at *m/z* 386, likely resulting from full sugar and CO₂ loss. A deeper fragment at *m/z* 251 indicated extensive breakdown of the aglycone structure between Ring C & D, supporting the identification of Zygophylloside S as a disaccharidic triterpenoid saponin. |
|  | **Compound (11)** |  |
| 1. 3-*O*-arabinosyl-quinovosyl quinovic acid | 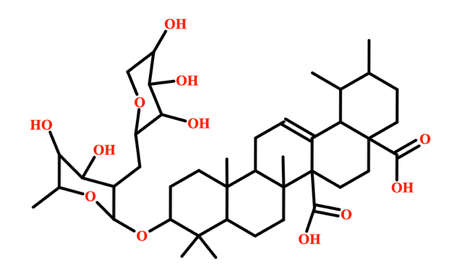 | In negative ion mode, 3-*O*-arabinosyl-quinovosyl quinovic acid exhibited a deprotonated molecular ion at *m/z* 763. The fragment at *m/z* 445 indicated the combined loss of the disaccharide moiety and CO₂, reflecting cleavage at both the C-3 glycosidic bond and the C-28 carboxyl group. The fragment at *m/z* 386 resulted from retro-Diels–Alder fragmentation of the aglycone, while the *m/z* 318 ion arose from further cleavage within the triterpenoid skeleton. These fragmentation pathways confirm the structure as a diglycosylated quinovic acid saponin with a free carboxylic acid. |
|  | **Compound (12)** |  |
| 1. Atriplicosaponin A | 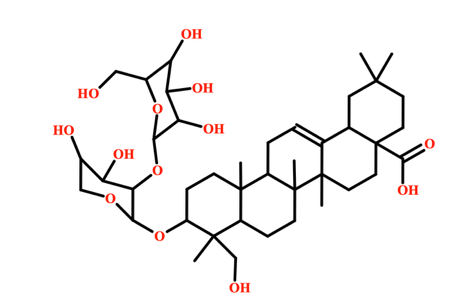 | Atriplicosaponin A exhibited a deprotonated molecular ion at *m/z* 765 in negative ion mode. The fragment at *m/z* 747 was attributed to dehydration, whereas *m/z* 721 indicated decarboxylation of the free C‑28 carboxyl group. The peak at *m/z* 603 reflected the loss of a hexose moiety, and progressive fragmentation produced *m/z* 545, likely due to additional neutral losses or sugar rearrangement. Aglycone fragments at *m/z* 288 and 221 corresponded to retro‑Diels–Alder cleavage and deeper degradation within the triterpenoid scaffold, respectively, confirming the presence of an ursane‑type aglycone. |
|  | **Compound (13)** |  |
| 1. Decarboxyquinovic acid-*O*-rhamnoside | 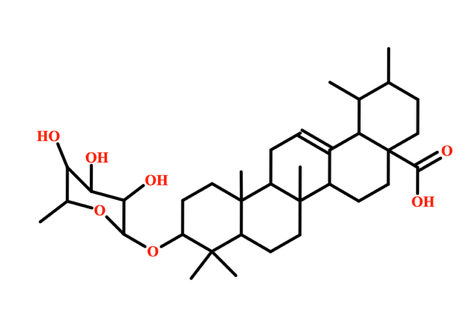 | In negative ion mode, decarboxyquinovic acid-*O*-rhamnoside showed a deprotonated molecular ion at *m/z* 587. MS/MS fragmentation yielded a daughter ion at *m/z* 381, corresponding to the loss of the rhamnosyl moiety and representing the free decarboxylated quinovic acid aglycone. A further fragment at *m/z* 261 was attributed to retro-Diels–Alder cleavage within the triterpenoid core, confirming the presence of an ursane-type skeleton. |
|  | **Compound (14)** |  |
| 1. Zygophyloside D | 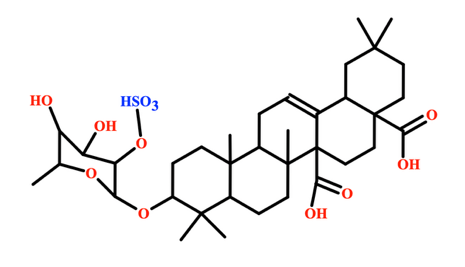 | Zygophyloside D displayed a deprotonated molecular ion at *m/z* 711 in negative ion mode. The fragment at *m/z* 667 indicated loss of CO_2_ from the free carboxyl group, while the ion at *m/z* 631 resulted from SO₃ elimination, suggesting a sulfated sugar moiety. The peak at *m/z* 485 corresponded to the aglycone ion following the loss of sulfo-deoxyhexose unit. Further fragmentation produced *m/z* 460, likely via decarboxylation or rearrangement, and a key aglycone-derived ion at *m/z* 251 attributed to retro-Diels–Alder cleavage between ring C & D, confirming the triterpenoid scaffold. |
|  | **Compound (15)** |  |
| 1. Quinovic acid-*O*-glucosyl ester | 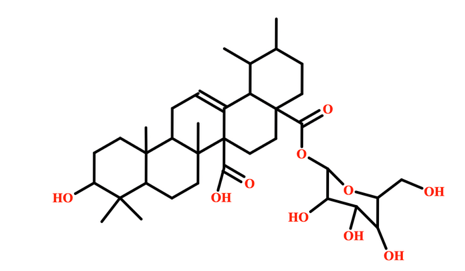 | In negative ion mode, quinovic acid‑*O‑*hexosyl ester showed a deprotonated molecular ion at *m/z* 647. The fragment at *m/z* 603 reflected loss of CO₂. A daughter ion at *m/z* 385 was attributed to elimination of the hexose unit together with CO₂, generating the free C‑3 hydroxyl quinovic acid aglycone. The fragment at *m/z* 317 indicates further breakdown of the triterpenoid skeleton, consistent with a quinovic acid‑type structure. |
|  | **Compound (16)** |  |
| 1. Zygophyloside I | 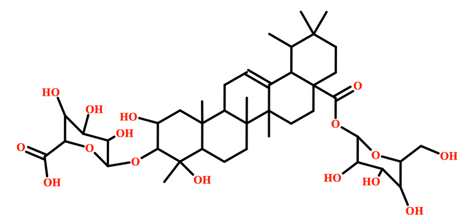 | Zygophylloside I showed a deprotonated molecular ion at *m/z* 825 in negative ion mode. Fragmentation yielded an ion at *m/z* 807, corresponding to dehydration, and an ion at *m/z* 781, arising from decarboxylation of the C‑28 carboxylic acid. A further fragment at *m/z* 487 indicated loss of the disaccharide moiety, confirming an O‑glycosidic linkage at C‑3 and a triterpenoid aglycone core. |
|  | **Compound (17)** |  |
| 1. *O*-quinovosyl cincholic acid-*O*-glucosyl ester | 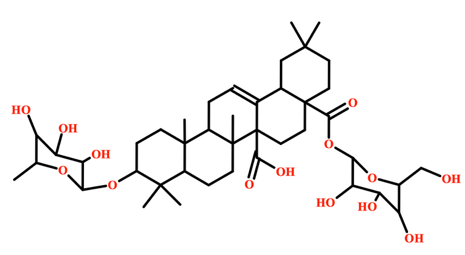 | In negative ion mode, *O*-quinovosyl cincholic acid-*O*-glucosyl ester exhibited a deprotonated molecular ion at *m/z* 793. Fragmentation yielded *m/z* 749, indicating decarboxylation of the C-28 carboxyl group. A fragment at *m/z* 631 corresponded to the cleavage of the ester-linked hexose, while *m/z* 317 was attributed to retro-Diels–Alder fragmentation between ring D & E of the triterpenoid aglycone. The ion at *m/z* 250 resulted from deeper fragmentation of the cincholic acid skeleton. |
|  | **Compound (18)** |  |
| 1. Methyl ester of Cincholic acid -*O-* glucoside | 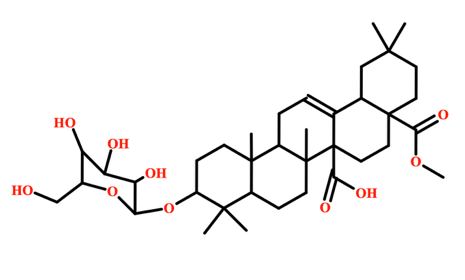 | In negative ion mode, methyl‑esterified cincholic acid‑*O*‑glucoside exhibited a deprotonated molecular ion at *m/z* 661. The fragment at *m/z* 558 reflected partial cleavage of the carboxyl and methyl ester group. A daughter ion at  *m/z* 499 indicated loss of the C‑3‑linked hexose, yielding the aglycone. The ion at *m/z* 329 was assigned to retro‑Diels–Alder cleavage within the triterpenoid backbone, supporting the structural assignment of an ursane‑type saponin with methyl esterification at C‑28. |
|  | **Compound (19)** |  |
| 1. Atriplicosaponin B | 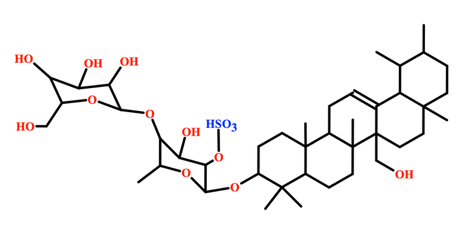 | In negative ion mode, Atriplicosaponin B exhibited a deprotonated molecular ion at *m/z* 829. Fragmentation yielded a daughter peak at *m/z* 342 attributed to retro-Diels–Alder cleavage within the triterpenoid backbone between rings D & E, following the loss of the two hexose sugars + SO_3_ + CH_2_OH |
|  | **Compound (20)** |  |
| 1. Cincholic acid -*O-* glucoside | 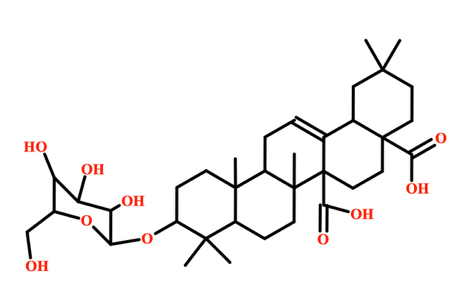 | In negative ion mode, cincholic acid-*O*-glucoside exhibited a deprotonated molecular ion at *m/z* 647. Fragmentation yielded an ion at *m/z* 603, attributed to the loss of CO₂ from the C-28 carboxylic group. A prominent fragment at *m/z* 317 corresponded to retro-Diels–Alder cleavage of the aglycone between rings D & E following deglycosylation and decarboxylation. The ion at *m/z* 251 was formed by further fragmentation of the triterpenoid skeleton, confirming the ursane-type structure of cincholic acid. |
|  | **Compound (21)** |  |
| 1. *O-*quinovosyl quinovic acid- quinovosyl ester | 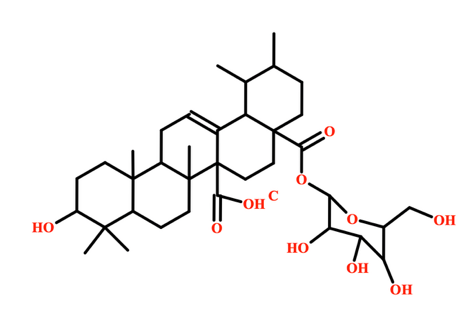 | In positive ion mode, *O*‑quinovosyl quinovic acid–quinovosyl ester exhibited a protonated molecular ion at *m/z* 779. The fragment at *m/z* 532 was attributed to loss of the ester‑linked quinovose unit at C‑28 together with CO₂, while the ion at *m/z* 381 arose from the combined loss of both sugar units and decarboxylation. A fragment at *m/z* 314 resulted from retro‑Diels–Alder cleavage between rings D and E within the quinovic acid aglycone, supporting the assignment of a doubly quinovosylated triterpenoid saponin. |
|  | **Compound (22)** |  |
| 1. Quinovic acid | 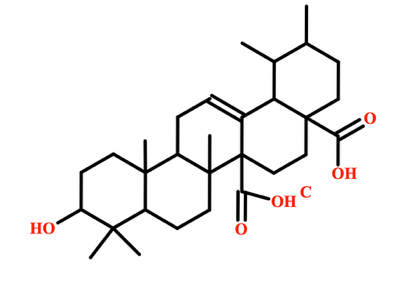 | In negative ion mode, quinovic acid produced a deprotonated molecular ion at *m/z* 485. The fragment at *m/z* 467 reflected the loss of water from the C-3 hydroxyl. A prominent fragment at *m/z* 251 resulted from retro-Diels–Alder cleavage between rings C & D of the triterpenoid core. Additional fragments at *m/z* 168 and 99 were attributed to deep aglycone fragmentation, confirming the ursane-type structure and typical fragmentation behaviour of quinovic acid. |
|  | **Compound (23)** |  |
| 1. Pomolic acid 3-*O-α*-L arabinoside | 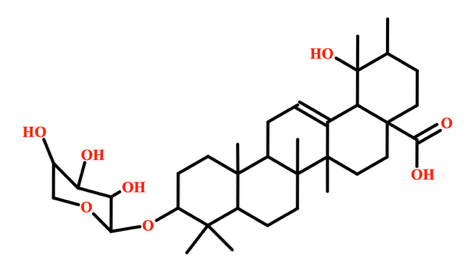 | In positive ion mode, pomolic acid 3-*O-α*-L-arabinoside exhibited a protonated molecular ion at *m/z* 605. Fragmentation yielded a prominent ion at *m/z* 471, corresponding to the loss of the arabinosyl unit, confirming the C-3 O-glycosidic linkage. The fragment at *m/z* 305 resulted from retro-Diels–Alder cleavage between ring B & C within the aglycone, providing structural confirmation of the pomolic acid backbone |
|  | **Compound (24)** |  |
| 1. Zygophyloside G | 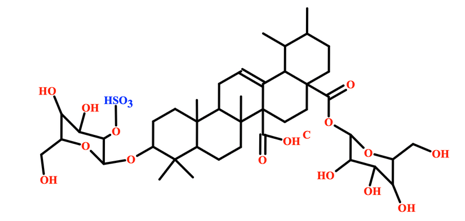 | Zygophyloside G showed a deprotonated molecular ion at *m/z* 889 in negative ion mode. The MS/MS fragment at *m/z* 548 indicated the loss of the sulphated sugar, followed by retro-Diels–Alder cleavage between ring A & B. A fragment at m/z 480 resulted from the subsequent retro-Diels–Alder cleavage between ring C & D of the aglycone. |
|  | **Compound (25)** |  |
| 1. Zygophylosides O | 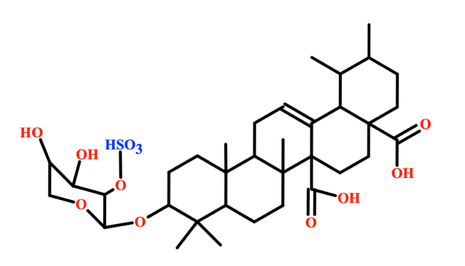 | Zygophylloside O showed a deprotonated molecular ion at *m/z* 697 in negative ion mode. The fragment at *m/z* 617 indicated neutral loss of SO₃, confirming a sulfated sugar moiety. A further fragment at *m/z* 379 arose from cleavage of the entire sulfated sugar unit together with two carboxyl groups, yielding the free aglycone. The ion at *m/z* 318 was attributed to retro‑Diels–Alder B/C‑ring cleavages within the triterpenoid skeleton, consistent with an ursane‑type aglycone. |
|  | **Compound (26)** |  |
| 1. 3-*O*-methoxy quinovic acid | 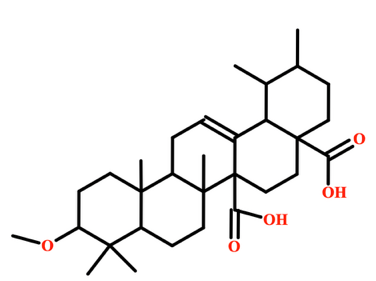 | In negative ion mode, 3‑*O*‑methoxy quinovic acid produced a deprotonated molecular ion at *m/z* 499. The fragment at *m/z* 455 indicated decarboxylation at C‑28. Ions at *m/z* 385 and 317 were consistent with sequential retro‑Diels–Alder cleavages between rings A/B and B/C of the aglycone, confirming an ursane‑type triterpenoid core. The fragment at *m/z* 249 reflected deeper skeletal fragmentation between rings C and D, further supporting its identification as a methylated quinovic acid derivative. |
|  | **Compound (27)** |  |
| 1. Methyl ester of 3-*O*-methoxy quinovic acid | 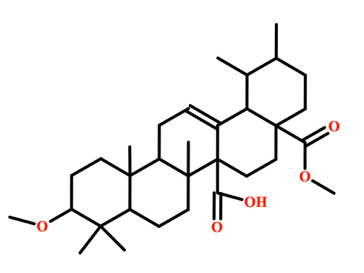 | In negative ion mode, methyl esterified 3-*O*-methoxy quinovic acid exhibited a deprotonated molecular ion at *m/z* 513. The fragment at *m/z* 469 indicated the loss of carbon dioxide from the C-28 methyl ester. The ion at *m/z* 249 was assigned to retro-Diels–Alder fragmentation between rings C & D of the aglycone, confirming an ursane-type triterpenoid skeleton. A fragment at *m/z* 114 likely resulted from cleavage of the methoxy group or side-chain rearrangement. |
|  | **Compound (28)** |  |
| 1. Cincholic acid -*O*- quinovoside | 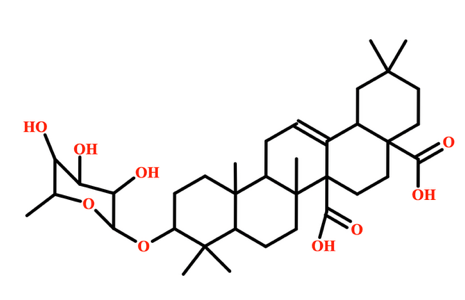 | In negative ion mode, cincholic acid-*O*-quinovoside produced a deprotonated molecular ion at *m/z* 631. A fragment at *m/z* 587 corresponded to decarboxylation of the C-28 carboxylic acid group. The ion at m/z 485 resulted from cleavage of the quinovosyl moiety, yielding the deprotonated cincholic acid aglycone. These fragments confirm the presence of a C-3-linked quinovose and a free carboxyl group, consistent with the proposed structure. |
|  | **Compound (29)** |  |
| 1. Methyl ester of Cincholic acid -*O*- quinovoside | 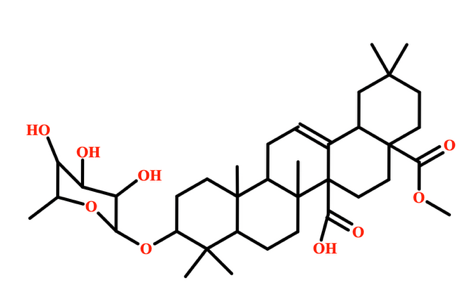 | In negative ion mode, methyl esterified cincholic acid-*O*-quinovoside exhibited a deprotonated molecular ion at *m/z* 645. The fragment at *m/z* 601 indicated the loss of carbon dioxide from the methyl ester at C-14. A second fragment at *m/z* 499 resulted from cleavage of the glycosidic bond with the quinovose sugar at C-3. The ion at *m/z* 313 was attributed to retro-Diels–Alder fragmentation between rings B & C of the triterpenoid core, confirming the ursane-type structure of cincholic acid and its substitution pattern. |
|  | **Compound (30)** |  |
| 1. Oleanolic acid | 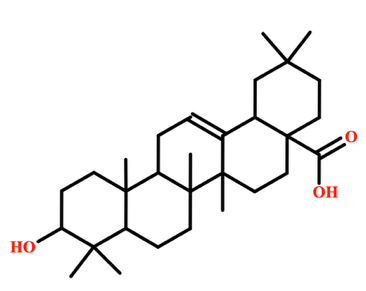 | In positive ion mode, oleanolic acid exhibited a protonated molecular ion at *m/z* 457. The fragment at *m/z* 412 resulted from decarboxylation at the C-28 carboxylic acid group. The ion at *m/z* 235 was assigned to retro-Diels–Alder cleavage between rings C & D of the oleanane-type triterpenoid core, while the *m/z* 168 fragment indicated deeper skeletal fragmentation. |
|  | **Compound (31)** |  |
| 1. Pomolic acid | 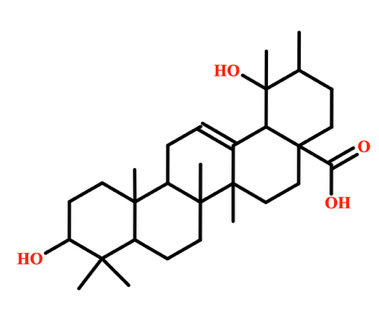 | In negative ion mode, pomolic acid exhibited a deprotonated molecular ion at *m/z* 471. Ms/Ms fragmentation yielded a product ion at *m/z* 453 due to loss of water from the C‑3 hydroxyl group. A fragment at *m/z* 427 corresponded to decarboxylation at C‑28. The fragment at *m/z* 235 was attributed to retro‑Diels–Alder cleavage of the triterpenoid skeleton, while the ion at *m/z* 100 resulted from deep aglycone fragmentation. These data confirm the ursane‑type structure of pomolic acid. |
|  | **Compound (32)** |  |
| 1. 14-decarboxyquinovic acid-*3β-O- β*-D-quinovosyl (l → 4)-quinovside | 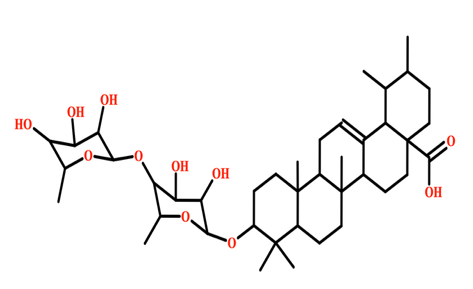 | In positive ion mode, 14-decarboxyquinovic acid-*3β-O-β-*D-quinovosyl-(1→4)-quinovoside exhibited a protonated molecular ion at *m/z* 735. Fragmentation yielded ions at *m/z* 691 (loss of a small neutral molecule), *m/z* 528 (cleavage of the terminal quinovose with rearrangement), and *m/z* 441 (loss of the full disaccharide moiety). The fragment at m/z 393 was assigned to retro-Diels–Alder fragmentation of the aglycone, confirming the ursane-type triterpenoid structure and the 3-O-disaccharide linkage. |
|  | **Compound (33)** |  |

Figure 1S: TLC plate with arrows pointing to crude saponins of *Z. decumbens* aerial parts, both A) before and B) after biocatalysis by *A. niger*.

***
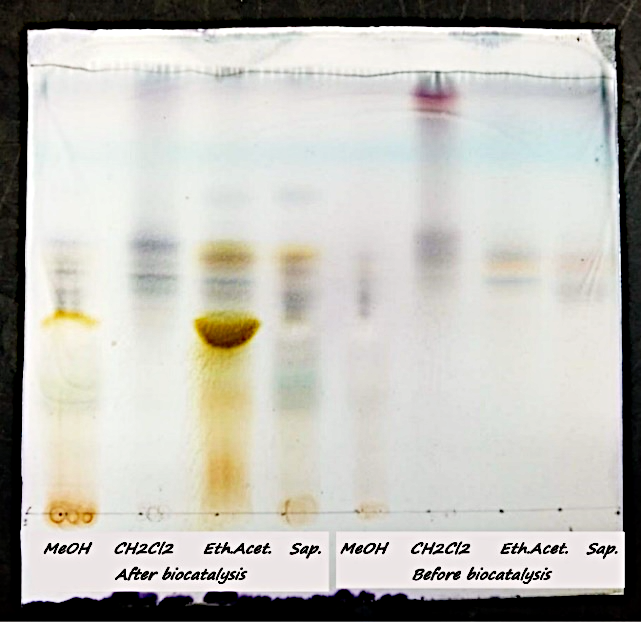
***

B

A

| **Figure 2S: Chromatogram of a) TS *versus* b) TSM of the aerial parts of *Z. decumbens* c) Blank in positive mode** |
| --- |
| 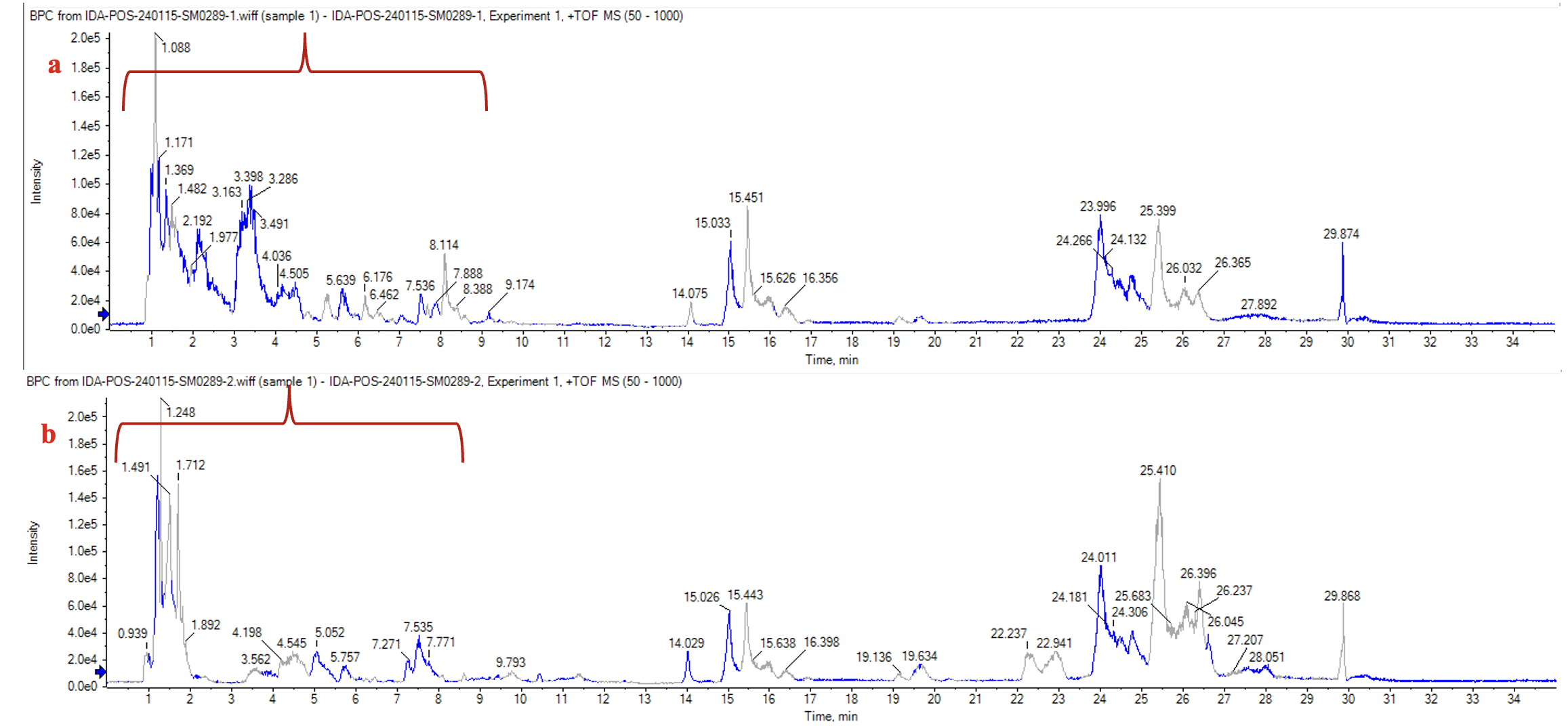 |
| 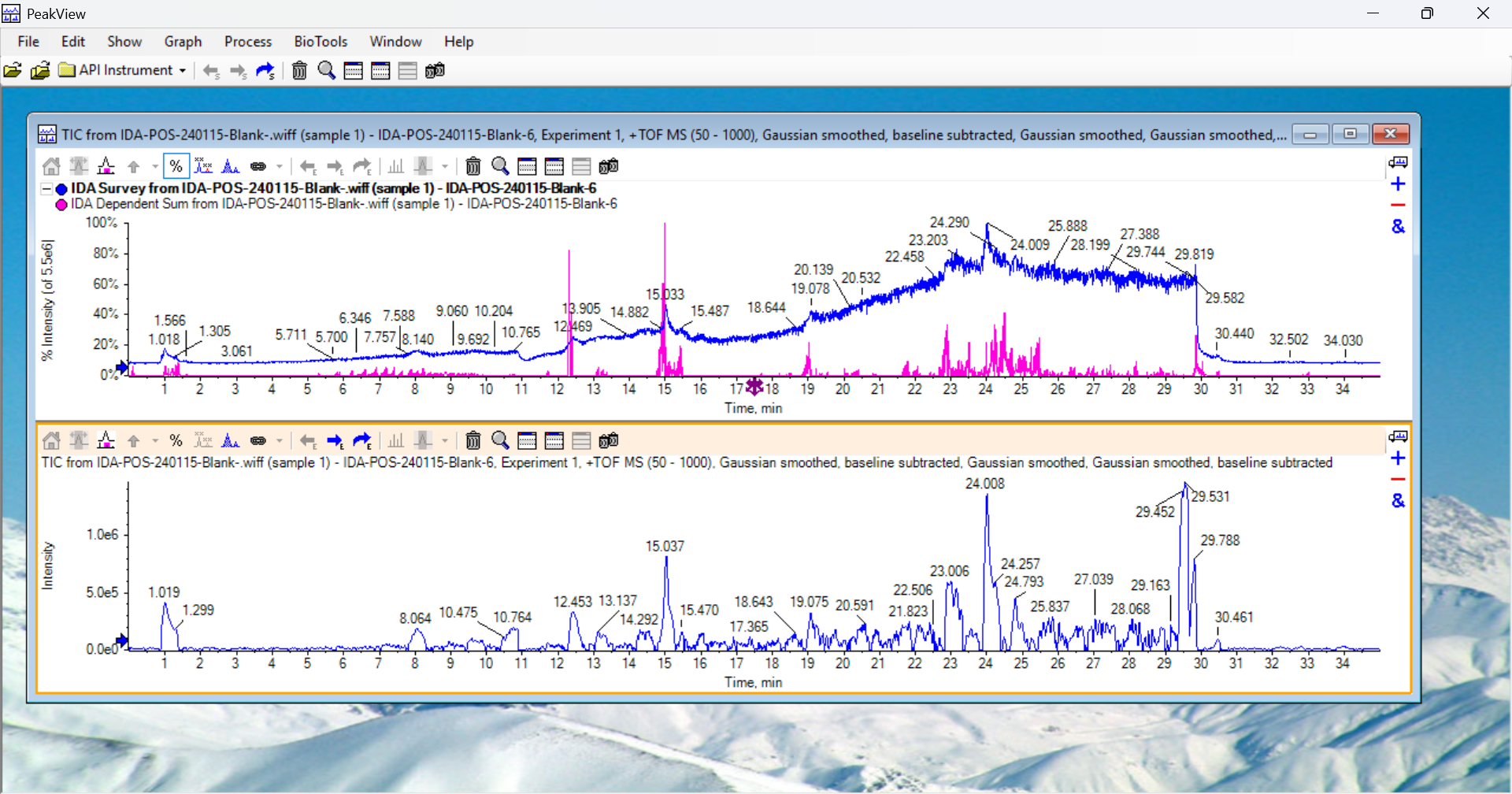  **C** |
| **Figure 3S: Chromatogram of a) TS, *versus* b) TSM of the aerial parts of *Z. decumbens* c) Blank in negative mode**  ****  **a**  **b**  **** |
| 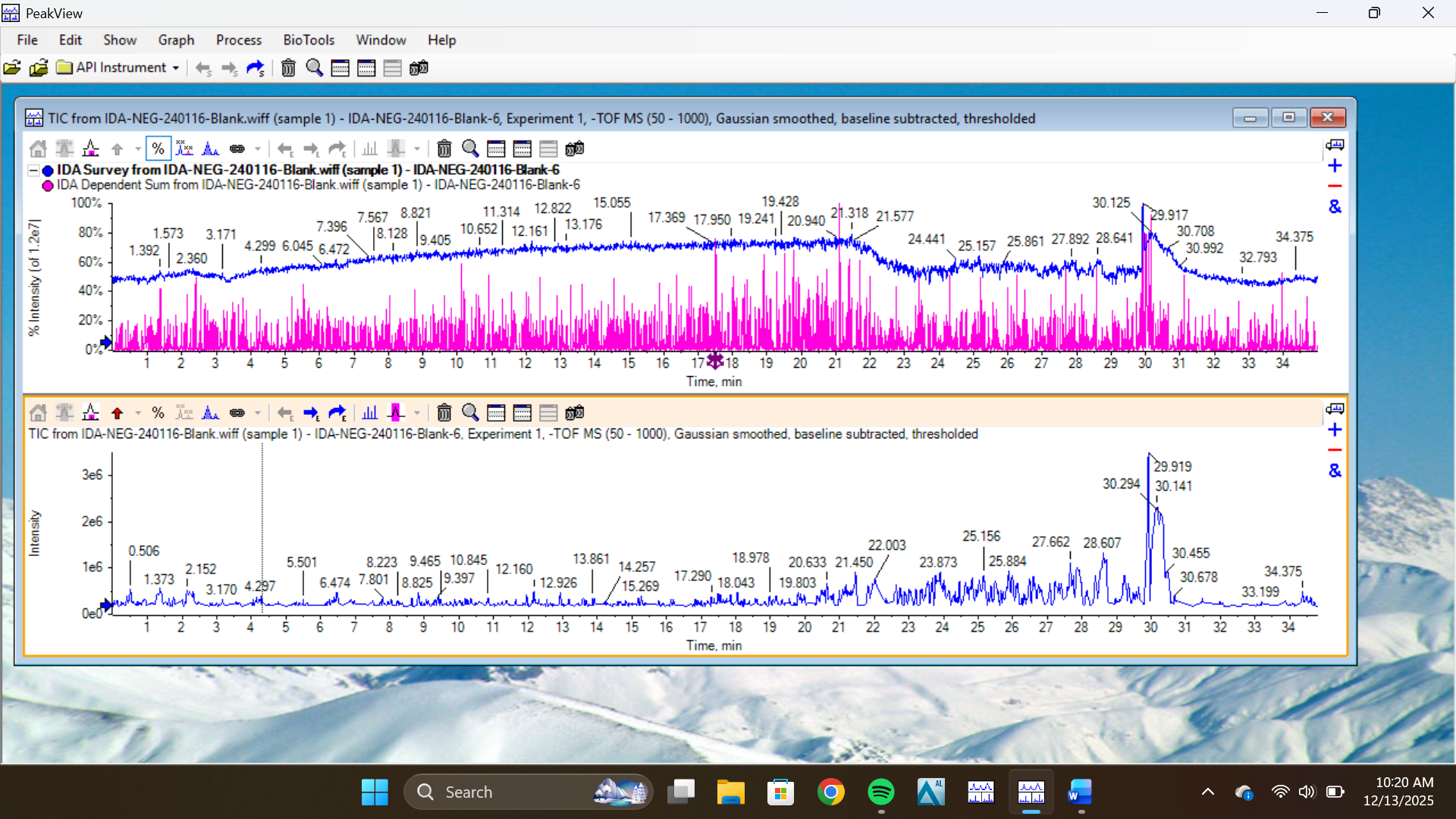  **C** |

**Figure 4S: LC/MS/MS of 28-*O*- *β* -D-glucoside ester of pomolic acid 3-*O*- *β* -D-2-*O* sulfonyl-glucoside of TSM in ESI-VE mode:**


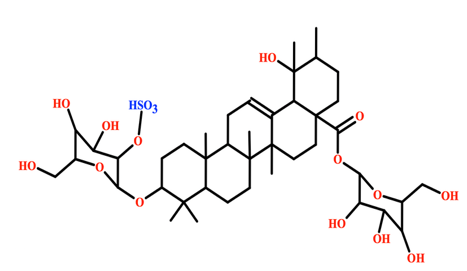


399

534

**Figure 5S: LC/MS/MS of Methyl ester of quinovic acid-*O*-glucosyl-rhamnoside of TSM in ESI-VE mode:**


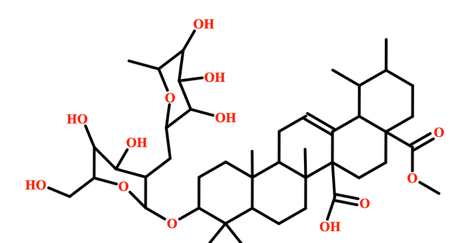


400

308

**Figure 6S: LC/MS/MS of quinovic acid-*O*-glucosyl ester of TSM in ESI-VE mode:**

**
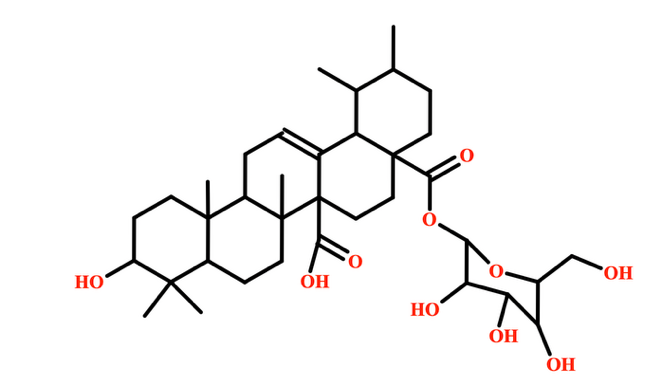
**

385

317

**Figure 7S: LC/MS/MS of Methyl ester of cincholic acid -*O-* glucoside of TSM in ESI-VE mode:**


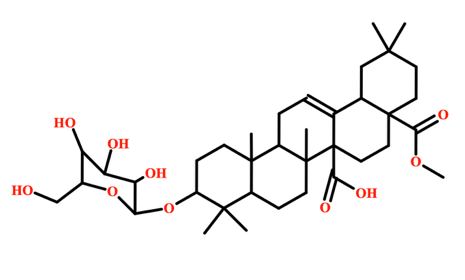


329

**Figure 8S: LC/MS/MS of Methyl ester of 3-*O*-methoxy quinovic acid of TSM in ESI-VE mode:**


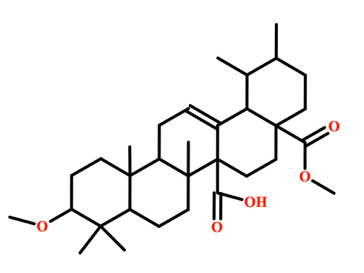


114

**Figure 9S: LC/MS/MS of 14-decarboxyquinovic acid-*3β-O- β*-D-quinovosyl (l → 4)-quinovside** **of TSM in ESI+VE mode:**


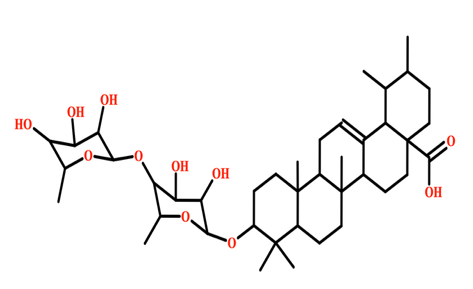


393

441

**Figure 10S: LC/MS/MS of Methyl ester of Cincholic acid -*O*- quinovoside of TSM in ESI-VE mode:**


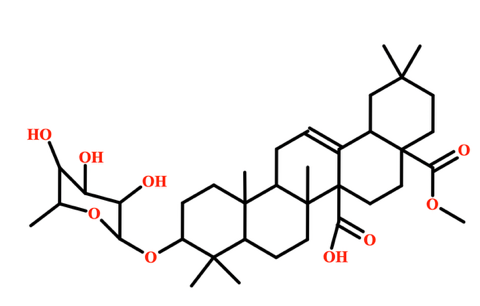


313

499

**Figure 11S: LC/MS/MS of Pomolic acid** **of TSM in ESI-VE mode:**


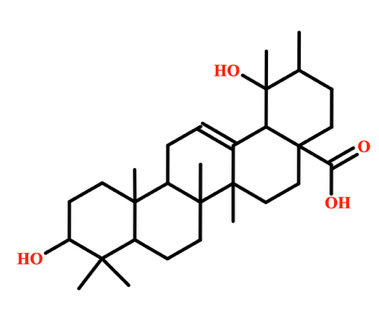


235

100

**Figure 12S: LC/MS/MS of Zygophyloside J of TS in ESI-VE mode:**

**
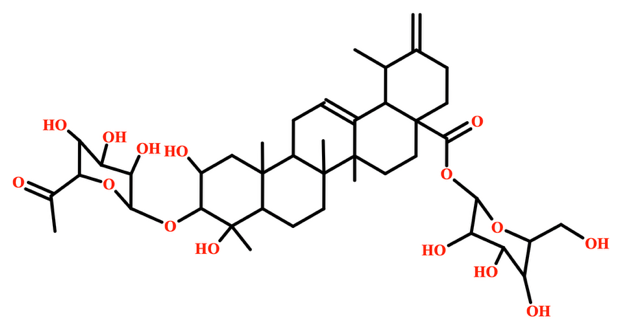
**

**Figure 13S: LC/MS/MS of 3-*O*- [*β*-D-(2-*O*- sulfonyl) glucosyl] quinovic acid of TS in ESI-VE mode:**

**
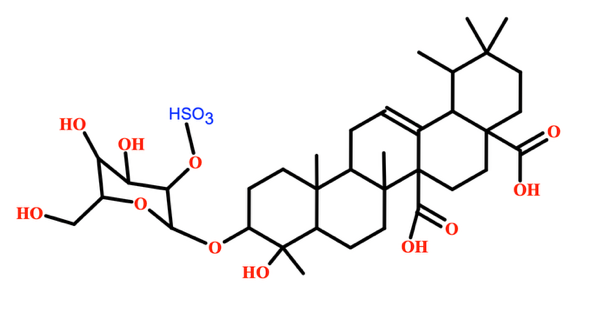
**

**Figure 14S: LC/MS/MS of Quinovic acid-*O*-glucosyl-rhamnoside of TS in ESI-VE mode:**


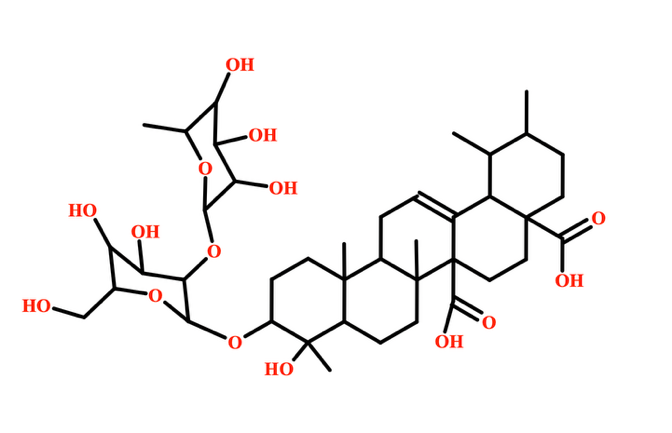


**Figure 15S: LC/MS/MS of Zygophyloside A of TS in ESI-VE mode:**

**
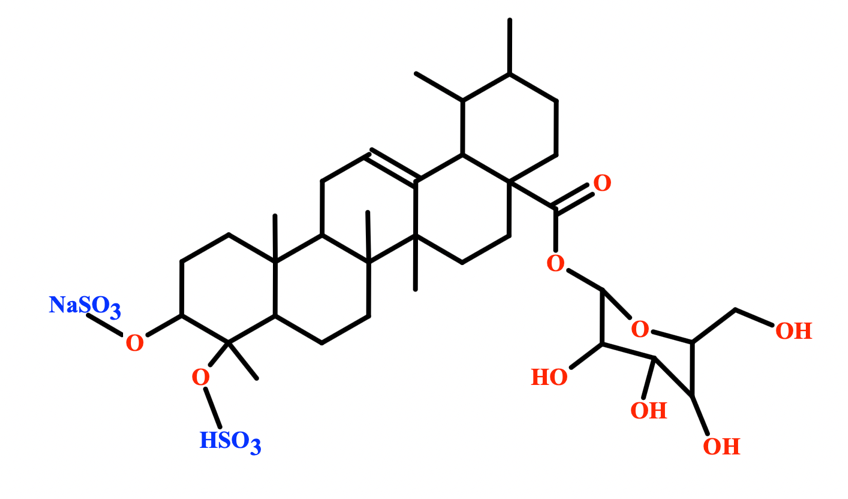
**

**Figure 16S: LC/MS/MS of 3-*β*-3-[(2-*O*-sulfo-*β*-D-en glucopyranurosonyl) oxy]-urs-20 -28-oic acid 28-(2-*O*-sulfo-*β*-D-glucosyl] ester of TS in ESI-VE mode:**


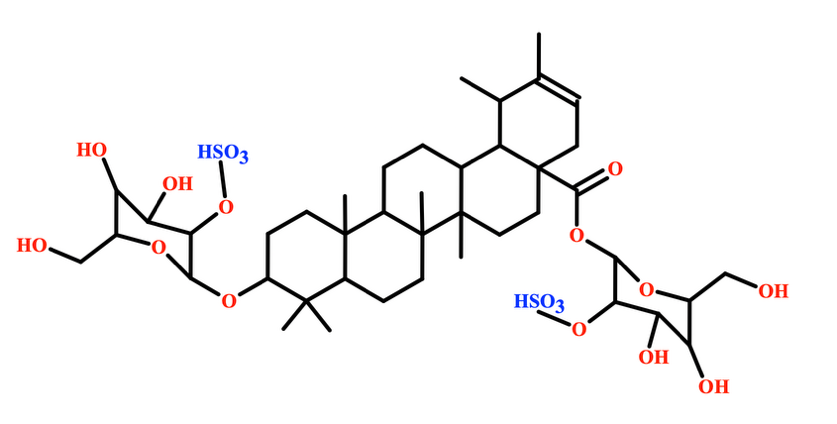


**Figure 17S: LC/MS/MS of 3-*O*-[*α*-L-arabinosyl (1→2)-*β*-D-glucosyl] quinovic acid 28- (*β*-D-glucosyl) ester of TS in ESI-VE mode:**


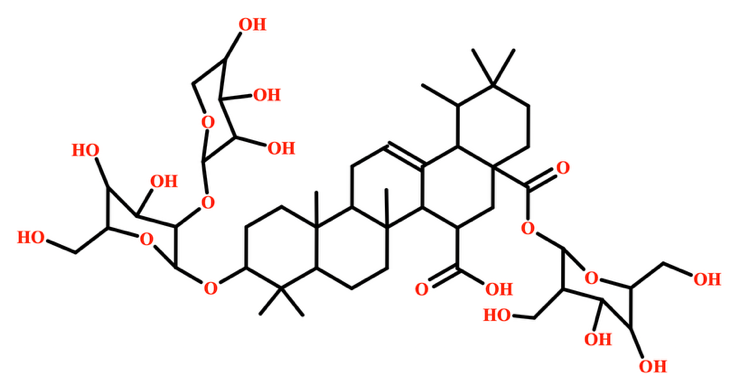


**Figure 18S: LC/MS/MS of 3-*O*-[*β*-D-glucosyl-(1→2) *β*-D-glucosyl] quinovic acid-28-*O-β*-D glucosyl ester of TS in ESI-VE mode:**


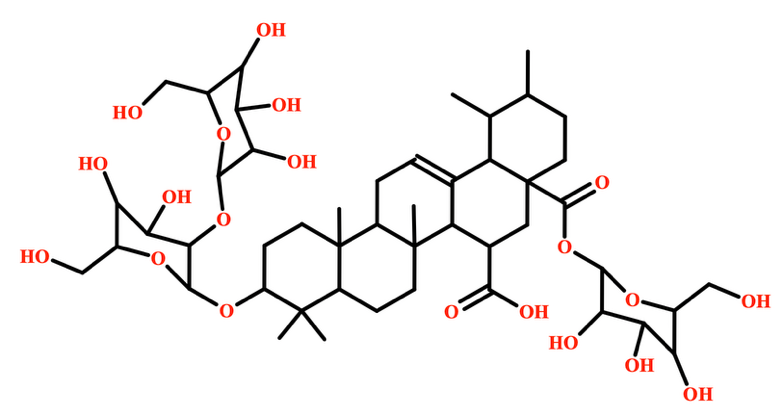


**Figure 19S: LC/MS/MS of Zygophyloside F of TS in ESI-VE mode:**


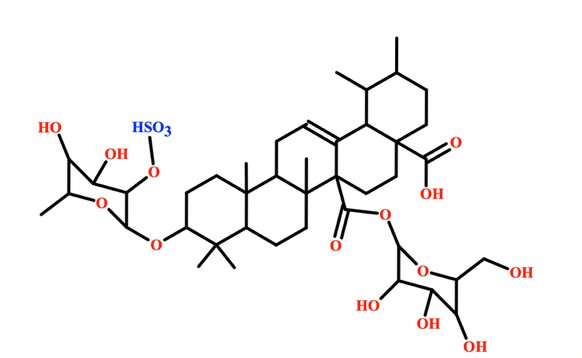


**Figure 20S: LC/MS/MS of Zygophyloside S of TS in ESI-VE mode:**


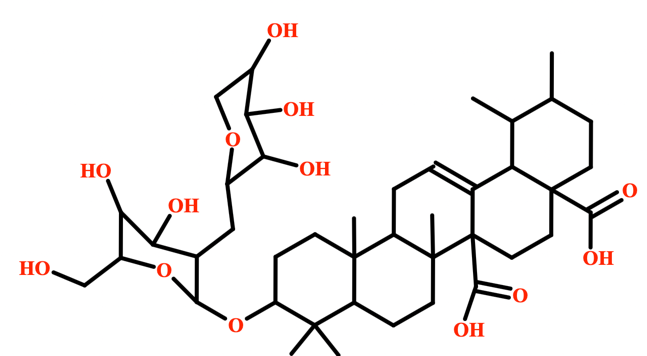


**Figure 21S: LC/MS/MS of 3-*O*-arabinosyl-quinovosyl quinovic acid of TS in ESI-VE mode:**

**
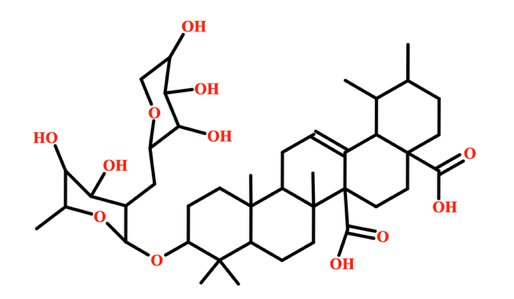
**

**Figure 22S: LC/MS/MS of Atriplicosaponin A of TS in ESI-VE mode:**

**
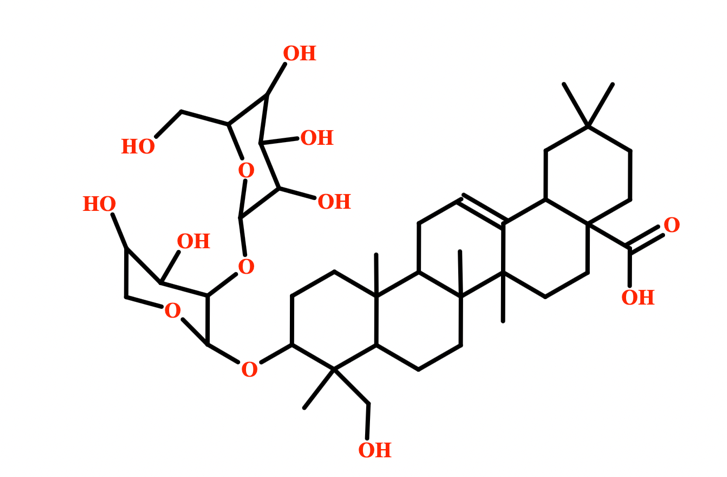
**

**Figure 23S: LC/MS/MS of Decarboxyquinovic acid-*O*-rhamnoside of TS in ESI-VE mode:**

**
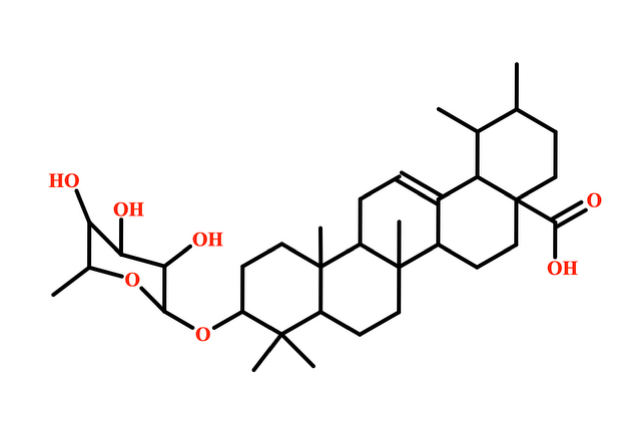
**

**Figure 24S: LC/MS/MS of Zygophyloside D of TS in ESI-VE mode:**

**
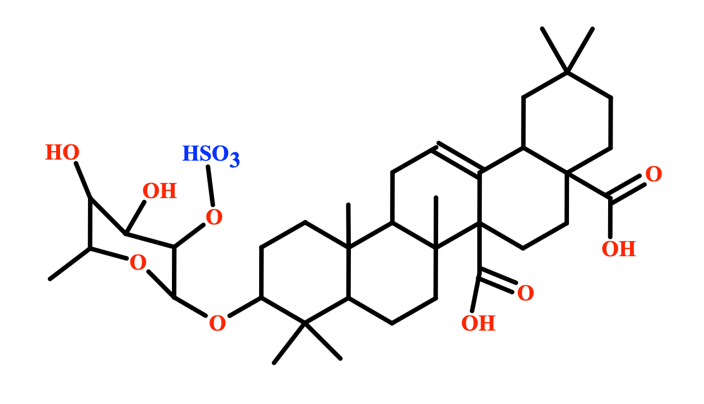
**

**Figure 25S: LC/MS/MS of Zygophyloside I of TS in ESI-VE mode:**

**
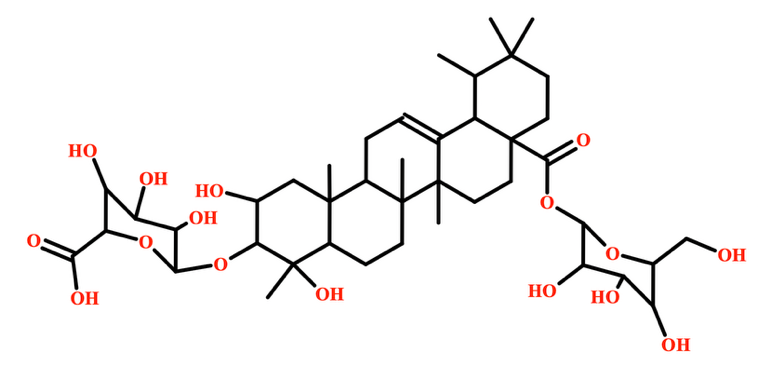
**

**Figure 26S: LC/MS/MS of *O*-quinovosyl cincholic acid-*O*-glucosyl ester of TS in ESI-VE mode:**

**
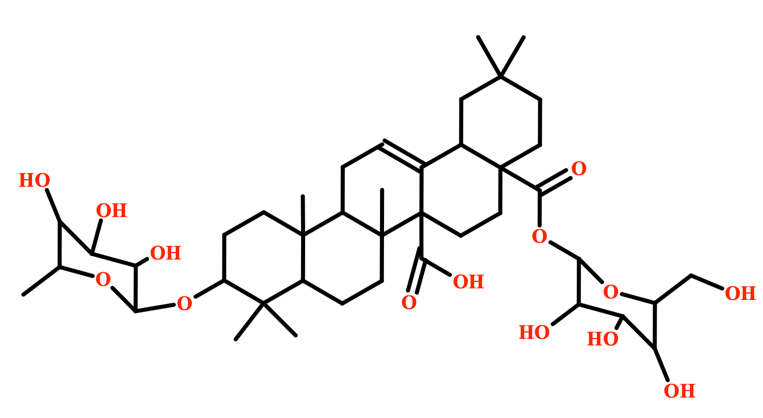
**

**Figure 27S: LC/MS/MS of Atriplicosaponin B of TS in ESI-VE mode:**

**
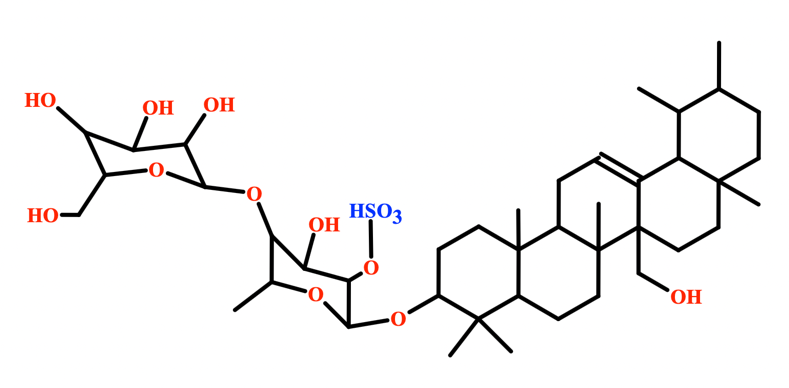
**

**Figure 28S: LC/MS/MS of Cincholic acid -*O*-glucoside of TS in ESI`-VE mode:**

**
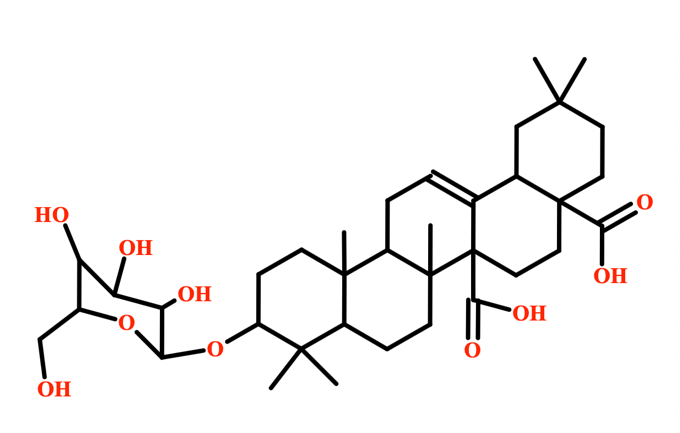
**

**Figure 29S: LC/MS/MS of *O*-quinovosyl quinovic acid – quinovosyl ester of TS in ESI+VE mode:**

**
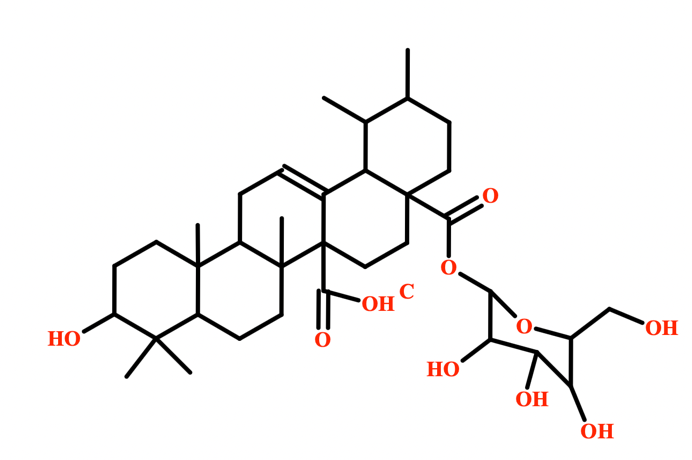
**

**Figure 30S: LC/MS/MS of Quinovic acid of TS in ESI-VE mode:**

**
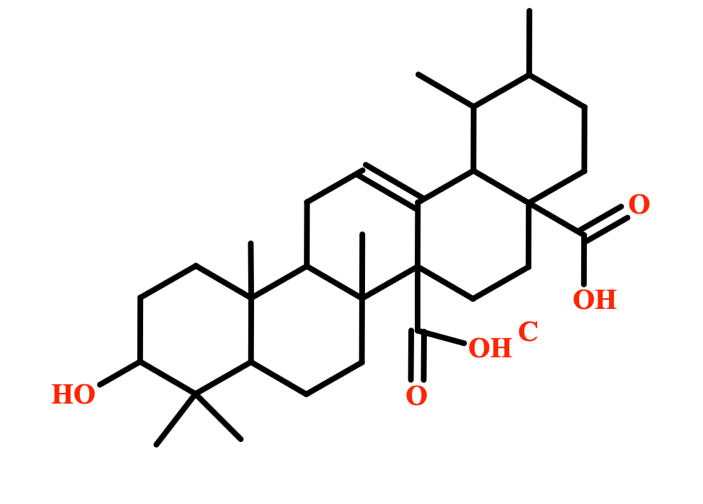
**

**Figure 31S: LC/MS/MS of Pomolic acid 3-*O*-*α*- L-arabinose of TS in ESI+VE mode:**

**
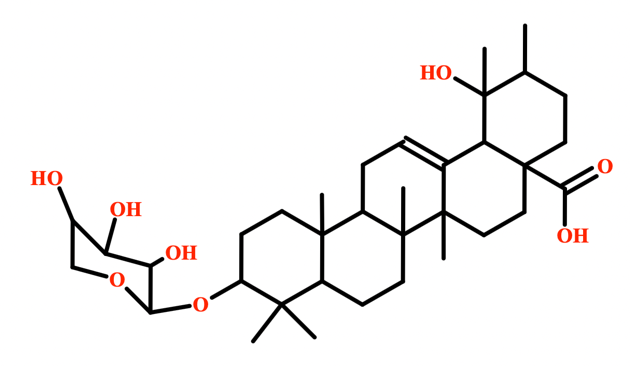
**

**Figure 32S: LC/MS/MS of Zygophyloside G of TS in ESI-VE mode:**


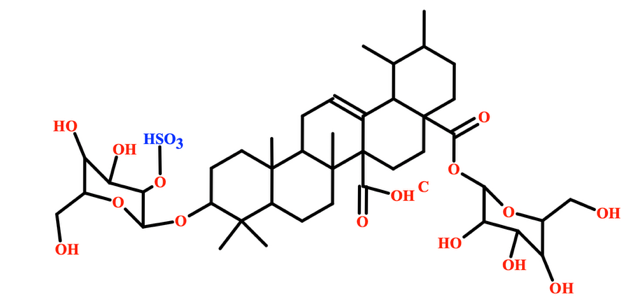


**Figure 33S: LC/MS/MS of Zygophyloside O of TS in ESI-VE mode:**


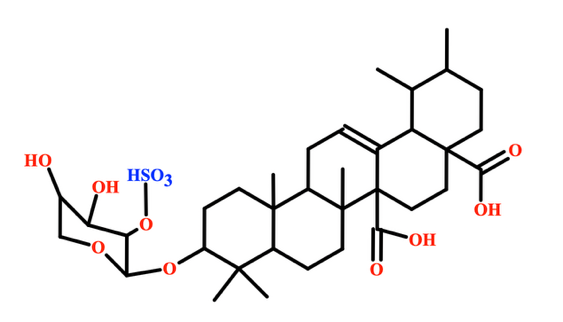


**Figure 34S: LC/MS/MS of 3-*O*- methoxy quinovic acid of TS in ESI-VE mode:**


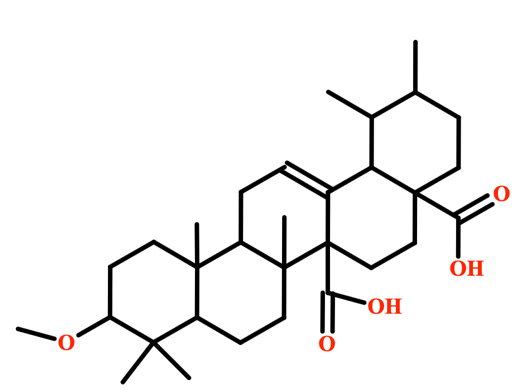


**Figure 35S: LC/MS/MS of Cincholic acid -*O-*quinovoside of TS in ESI-VE mode:**


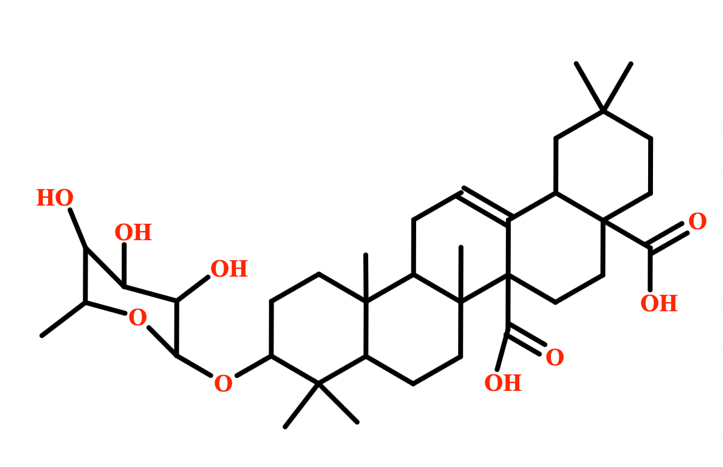


**Figure 36S: LC/MS/MS of Oleanolic acid of TS in ESI+VE mode:**


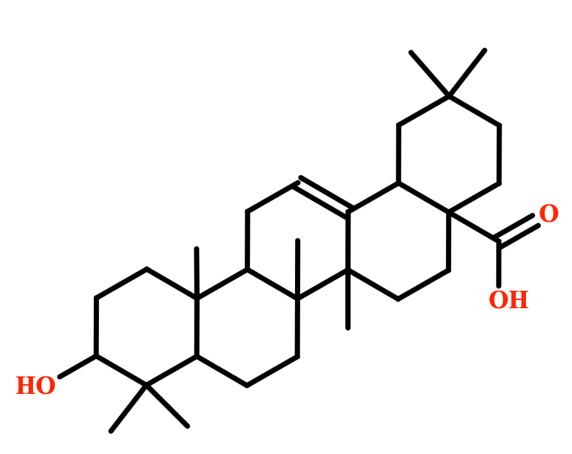


**Figure 37S: Microscope images (X100) showing the effect of 25, 50 & 100µg/mL concentrations of a) TS, b) TSM of *Zygophyllum decumbens,* and c) Negative control on HepG2, HT-29, and BNLCL2 cell lines*:***

| **HepG-2 cell lines** | | | |
| --- | --- | --- | --- |
| **a)TS** | 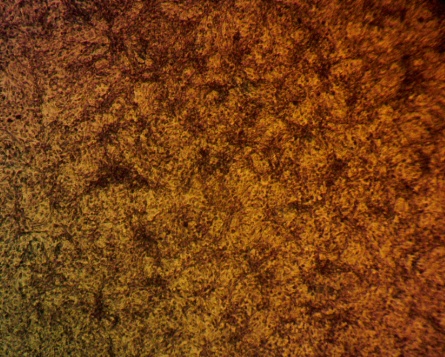 | **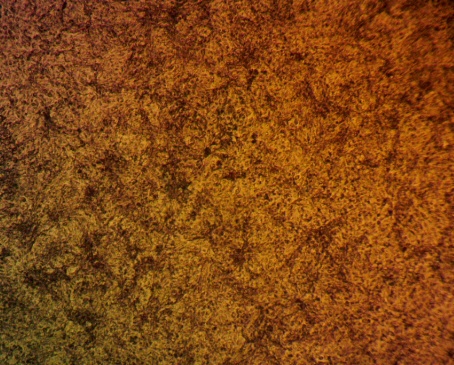** | **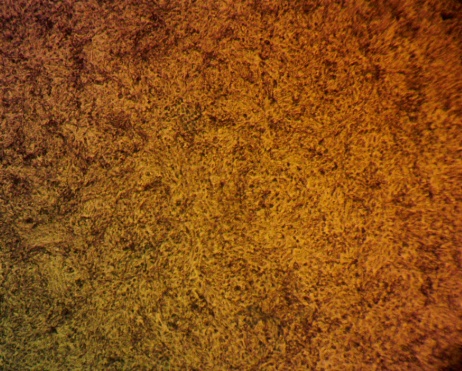** |
|  | **Conc.** **25µg/mL** | **Conc.** **50µg/mL** | **Conc.** **100µg/mL** |
| **b)TSM** | 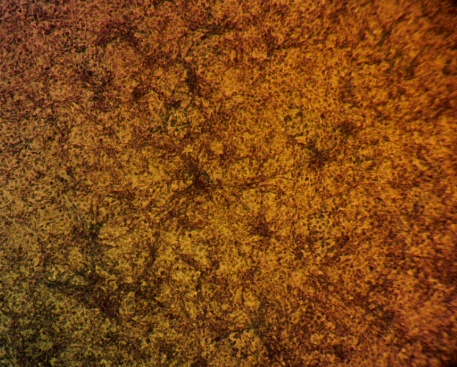 | **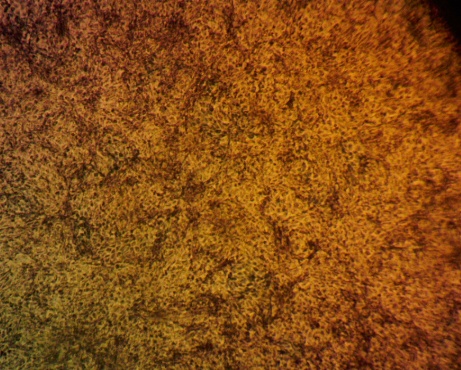** | **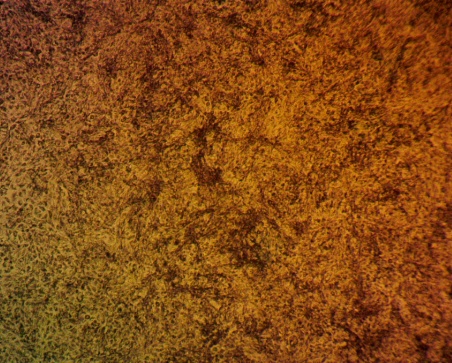** |
|  | **Conc.** **25µg/mL** | **Conc.** **50µg/mL** | **Conc.** **100µg/mL** |
| **c)Control** | **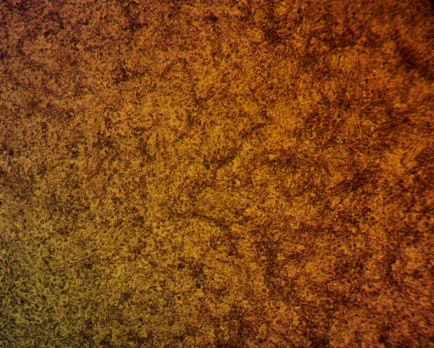** | | |
| **HT-29 cell lines** | | | |
| **a)TS** | 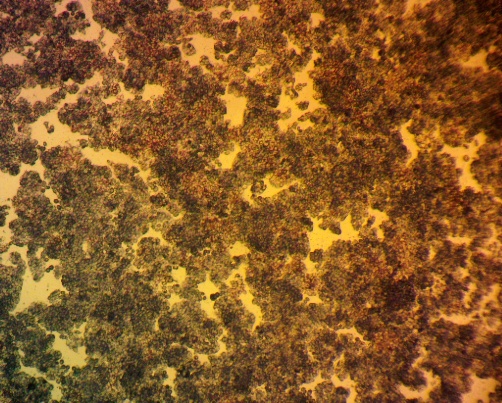 | **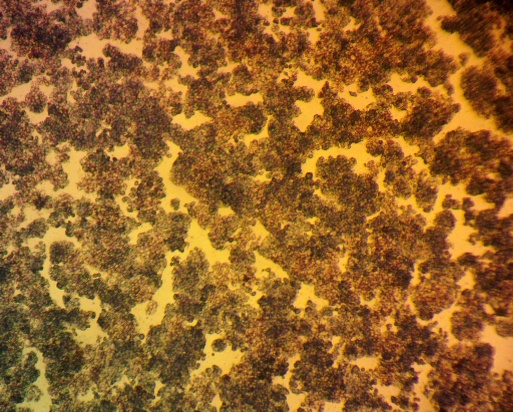** | **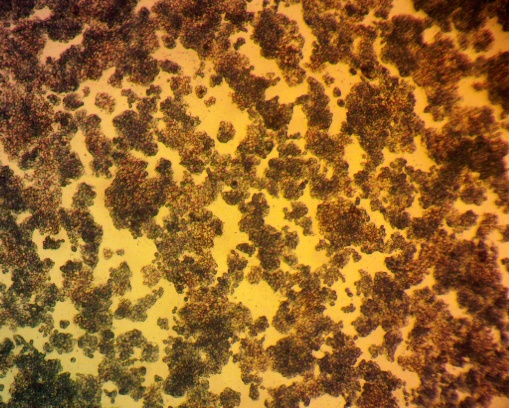** |
|  | **Conc.** **25µg/mL** | **Conc.** **50µg/mL** | **Conc.** **100µg/mL** |
| **b)TSM** | 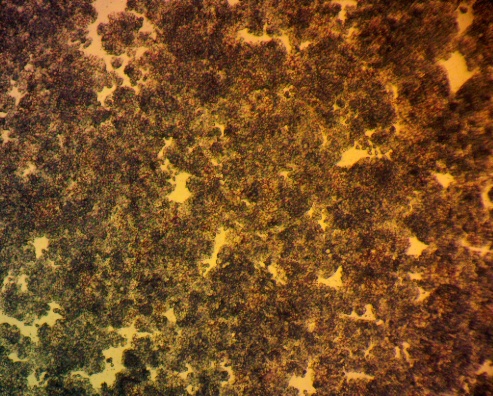 | **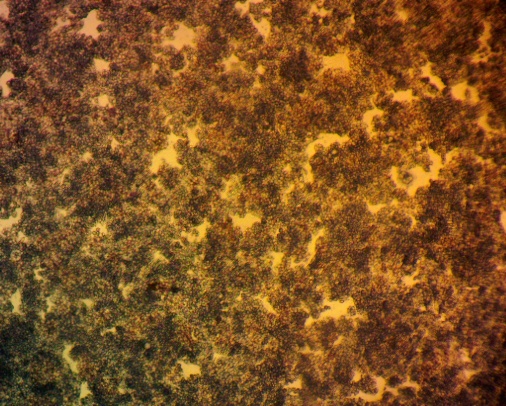** | **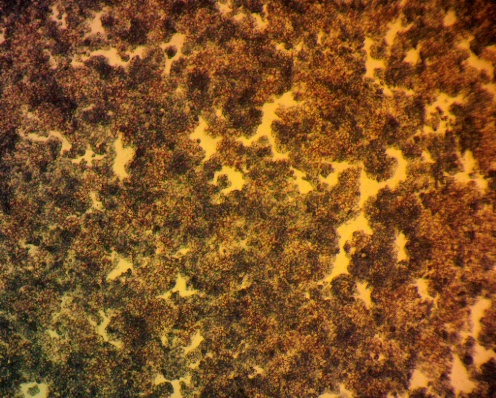** |
|  | **Conc.** **25µg/mL** | **Conc.** **50µg/mL** | **Conc.** **100µg/mL** |
| **c)Control** | 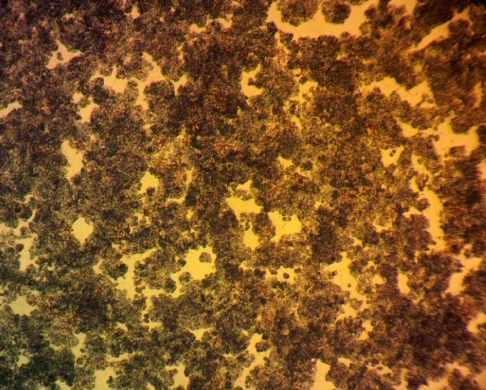 | | |
| **BNL CL2 cell lines** | | | |
| **a)TS** | 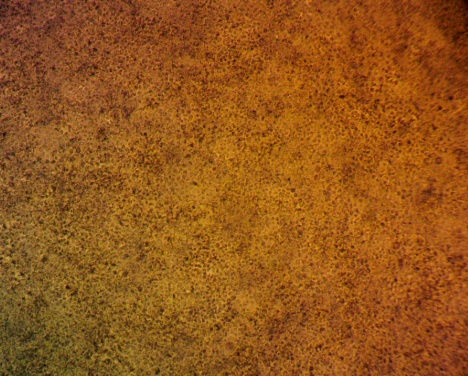 | **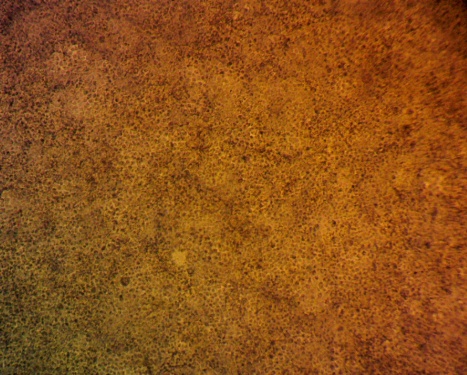** | **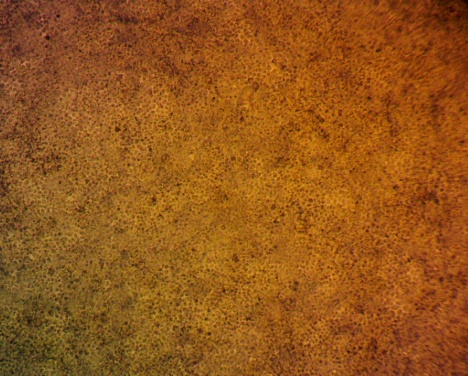** |
|  | **Conc.** **25µg/mL** | **Conc.** **50µg/mL** | **Conc.** **100µg/mL** |
| **b)TSM** | 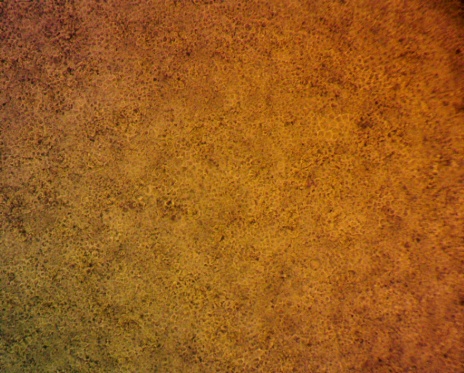 | **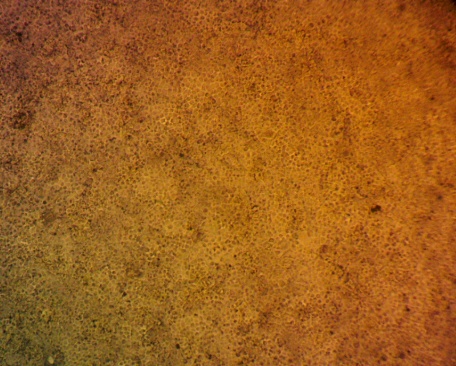** | **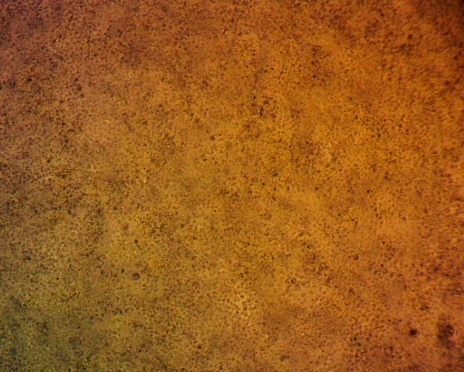** |
|  | **Conc.** **25µg/mL** | **Conc.** **50µg/mL** | **Conc.** **100µg/mL** |
| **c)Control** | **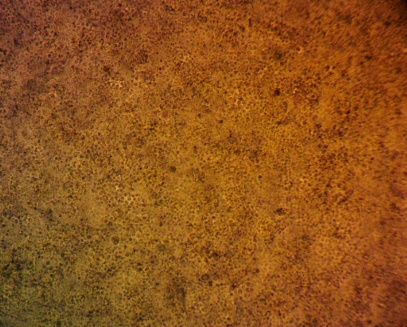** | | |

**Figure 38S: Suggested biocatalytic mechanisms that led to the emergence of eight unique rare saponins** **in the TSM of *Zygophyllum decumbens***

***
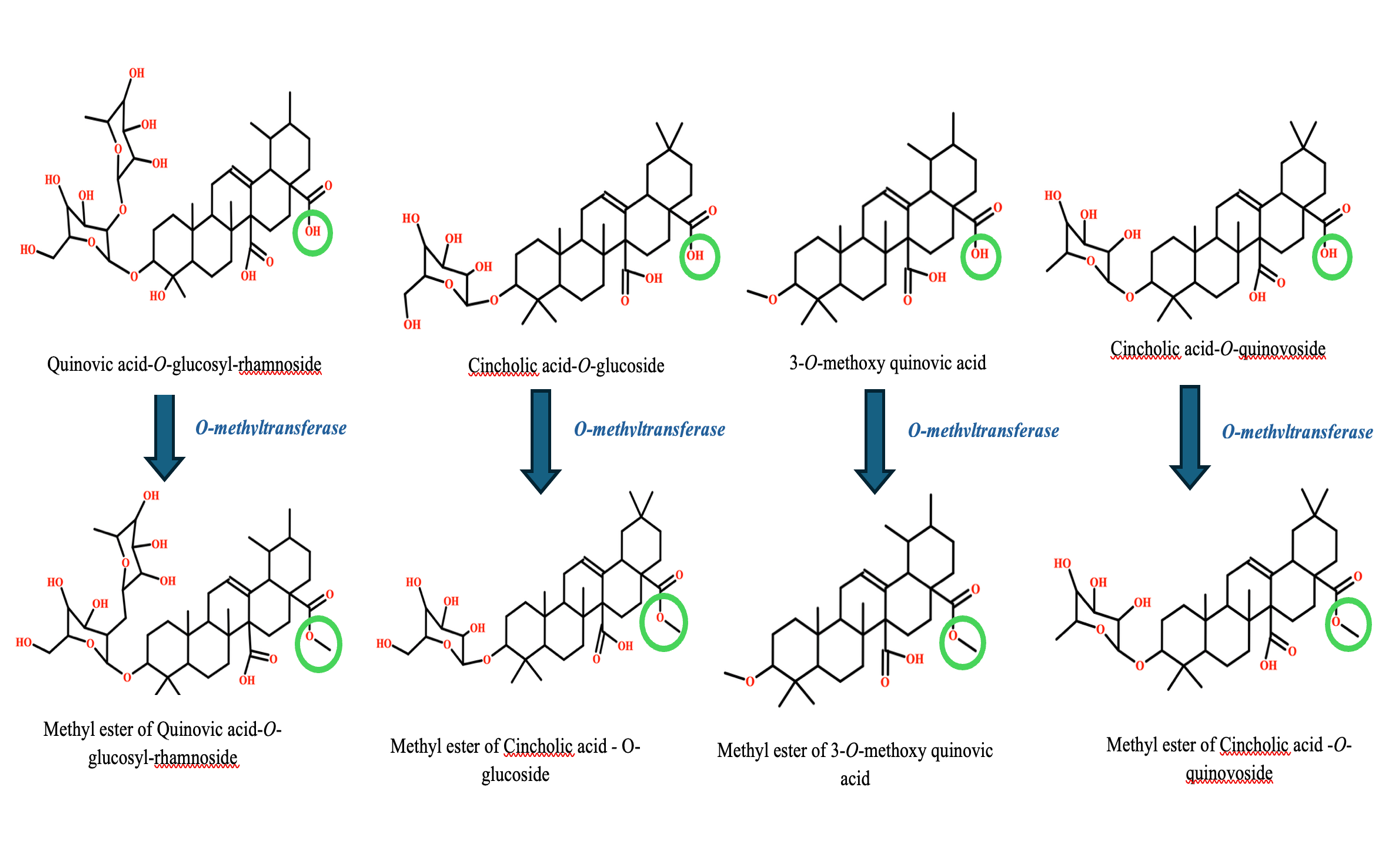

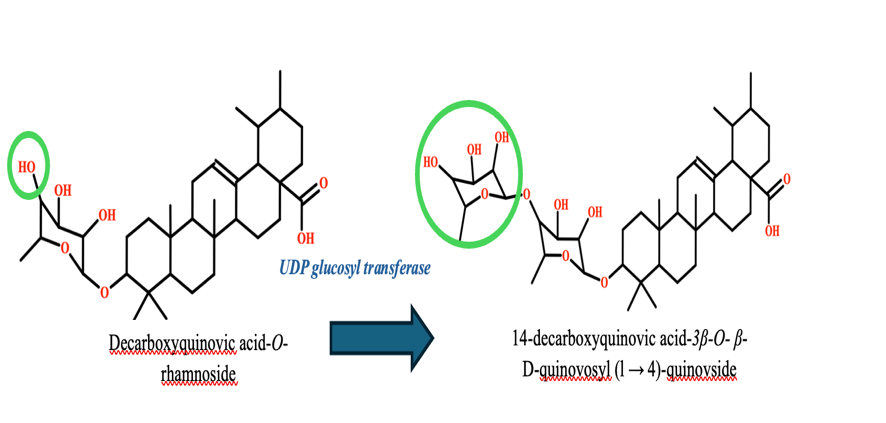
***

**B**

**A**


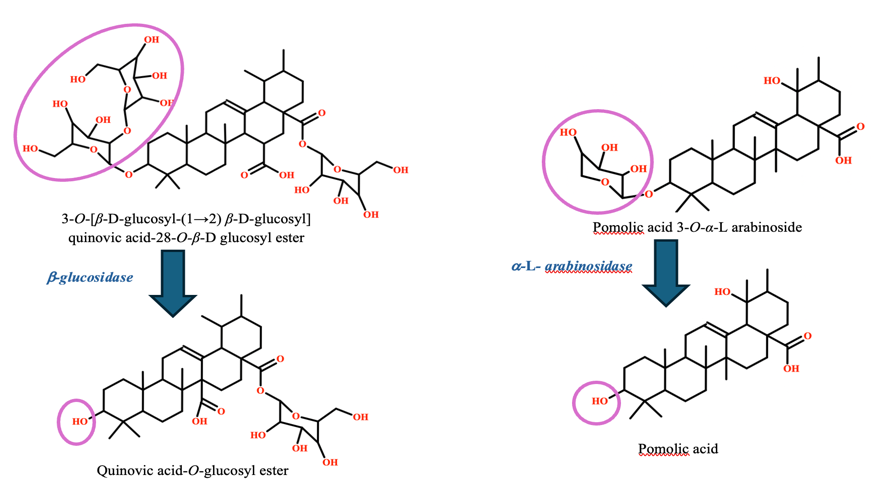


**C**


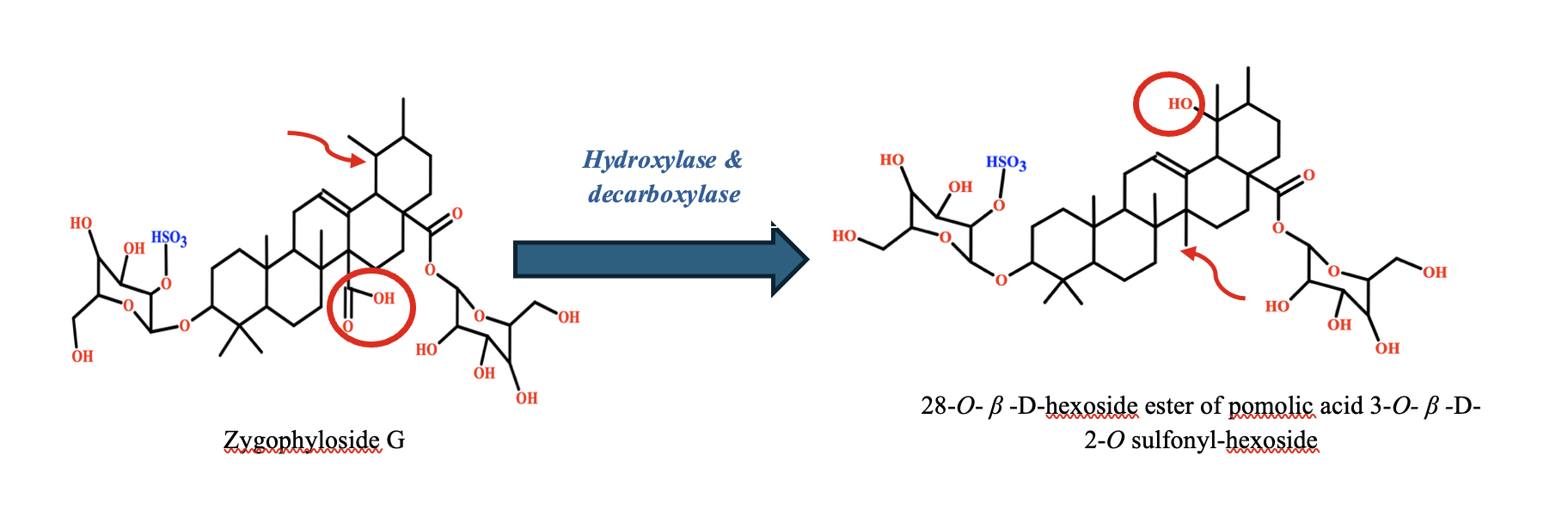


**D**
